# Binding of group 15 and group 16 oxides by a concave host containing an isophthalamide unit

**DOI:** 10.3762/bjoc.8.2

**Published:** 2012-01-03

**Authors:** Jens Eckelmann, Vittorio Saggiomo, Svenja Fischmann, Ulrich Lüning

**Affiliations:** 1 Otto-Diels-Institut für Organische Chemie, Christian-Albrechts-Universität zu Kiel, Olshausenstr. 40, D-24098 Kiel, Germany

**Keywords:** anion binding, association constant, estimation of binding constants, macrocycle, molecular recognition

## Abstract

A bi-macrocycle with an incorporated isophthalamide substructure was synthesized by double amide formation between an isophthaloyl dichloride and two equivalents of a bis(alkenyloxy)aniline, followed by ring-closing metathesis and hydrogenation. In contrast to many related isophthalamides, the concave host exhibits a better binding for oxides, such as DMSO or pyridine-*N*-oxide, than for halide anions. A general method for a quick estimation of the strength of binding derived from only a few data points is presented and gives an estimated *K*_ass_ of pyridine-*N*-oxide of ca. 40 M^−1^, NMR titration confirms 25 M^−1^.

## Introduction

**Figure 1:**
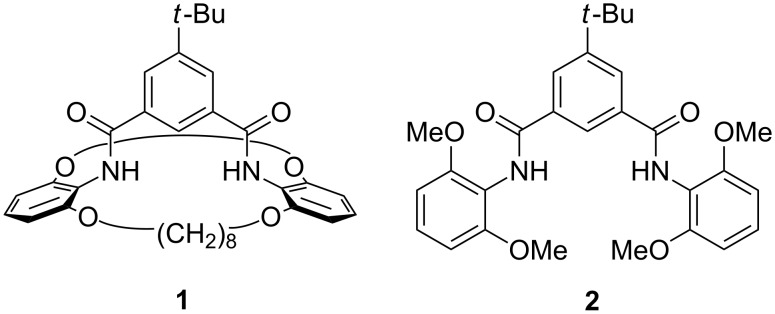
Bi-macrocyclic concave host **1** and its non-macrocyclic model **2**.

In the last decade, isophthalamide derivatives have become attractive neutral hosts as anion receptors [1–2]. Some of these derivatives show a high selectivity for one anion over others [3]. Isophthalamide units have also been incorporated into macrocycles [4–5] or bi-macrocycles for ion-pair and ion-triplet recognition [6–9]. During the last few years, it was also shown that the orientation of the amide bonds of the isophthalamides plays an important role in the effectiveness of anion binding and subsequently in applications such as transmembrane anion transport. Rotation along the amide–aryl bonds leads to *syn/anti*, *syn/syn* and *anti/anti* conformers (*syn* and *anti* defined with respect to the relative orientation of the NH atoms), and only the *syn/syn* conformer of an isophthalamide is capable of simultaneously binding an anion by *both* NH groups. The *syn/syn* conformation can be stabilized by using isophthalamide derivatives capable of intramolecular hydrogen bonding to the CO part of the amide groups [10–11], or by other means of bridging [12]. Due to the preorganization of such molecules, the binding constant for chloride is impressively increased with respect to the non-preorganized isophthalamides. However, an intramolecular hydrogen bond can be easily broken in polar solvents, hence destroying the preorganization and thus decreasing the binding affinities for the anions. Herein we describe the facile synthesis and the binding properties of a concave host **1** with a different type of preorganization. This contains an isophthalamide unit in a bi-macrocyclic structure (Figure 1). Concave hosts and especially concave reagents are best envisioned as having the form of a lamp in a lampshade in which the light bulb is the reactive centre [13–15]. The preorganization and the exact shape of the “lampshade” determine the selectivity and the difference in binding of various guests.

## Results and Discussion

### Synthesis

**Scheme 1:**
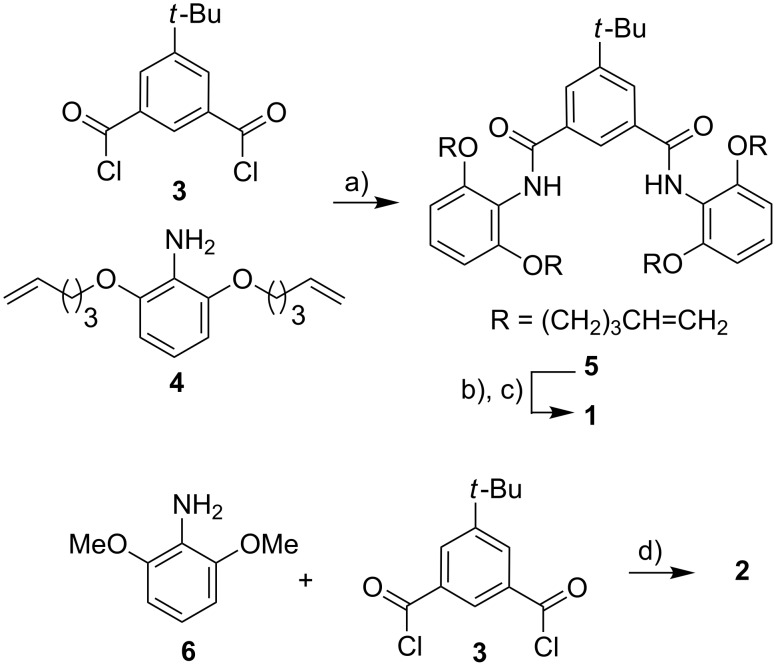
Synthetic scheme for the syntheses of concave host **1** and non-macrocyclic derivative **2**. a) Et_3_N, THF, 16 h, room temp., 61%; b) Grubbs 1st gen. cat., CH_2_Cl_2_, 24 h, room temp.; c) Pd/C, H_2_, MeOH, 24 h, room temp., 77%; d) Et_3_N, THF, 18 h, room temp., 97%.

Besides the desired bi-macrocycle **1**, isophthalamide **2** was synthesized in order to compare the binding properties of a non-macrocyclic host with the concave host **1**. The syntheses of the concave host **1** and its analogue **2** are depicted in Scheme 1.

The preparation of concave host **1** starts with aniline **4**, which was synthesized as previously published [16]. The reaction of two equivalents of this aniline **4** with isophthaloyl dichloride **3** gave the open diamide **5**. This tetraalkene **5** was then converted to bi-macrocycle **1** by ring-closing metathesis followed by catalytic hydrogenation. Model compound **2** was obtained by reacting isophthaloyl dichloride **3** with 2,6-dimethoxyaniline (**6**). The two products **1** and **2** were isolated and characterized. The preorganization of the binding region was investigated by NOESY experiments. While two cross peaks between the NH protons and both types of protons in the central aromatic region were observed in the case of the more flexible compound **2**, there was only one cross peak of an aromatic proton of the isophthalamide of bi-macrocycle **1** with the NH protons: The *endo*-proton in the 2-position of the isophthalic unit is in close proximity to the NH protons. Thus, the binding region of **1** is preorganized (for details see Supporting Information File 1).

### NMR binding studies

**Figure 2:**
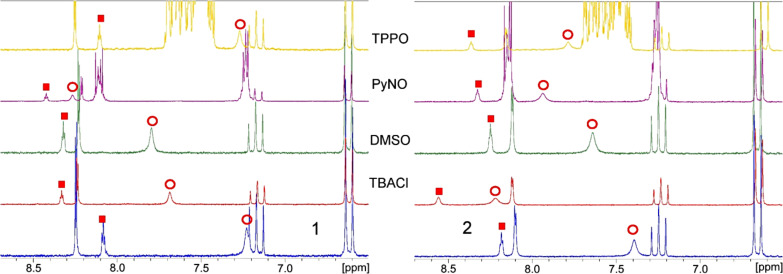
Expansion of a part of the ^1^H NMR spectra (200 MHz, 298 K) of pure **1** and **2** in CD_2_Cl_2_ (bottom) and after addition of TBACl, DMSO, pyridine-*N*-oxide (PyNO), triphenylphosphine oxide (TPPO), from bottom to top, respectively. NH proton (red circles), *endo*-CH proton (red squares).

Each of the isophthalamides, **1** and **2**, was dissolved in CD_2_Cl_2_ and ^1^H NMR spectra were recorded after addition of five equivalents of different tetrabutylammonium halide salts (TBAHal). The chemically induced shifts (CIS) of the NH and the isophthalamide *endo*-CH protons (i.e., the 2-position of the aromatic ring) were analyzed (Figure 2). Further addition of guests led to larger CIS, but no saturation was observed. The shallow curvature and absence of saturation suggest small binding constants. The different magnitudes of the CIS suggest that concave host **1** binds halides with lower affinity than its acyclic relative **2**, although the magnitude of the CIS need not be correlated with the binding constants.

However, when sulfoxides were added as neutral guests, the relative binding of these guests by **1** and **2** showed drastic differences. Dimethyl sulfoxide (DMSO), methylphenyl sulfoxide (MPSO) and diphenyl sulfoxides (DPSO) induced a shift of the *endo*-CH in the concave host **1** of 0.24 ppm, 0.20 ppm and 0.25 ppm, respectively (for DMSO see Figure 2, left, see also Supporting Information File 1), while the addition of these guests had almost no effect on the *endo*-CH of model compound **2** (for DMSO see Figure 2, right). Although there is almost no CIS observed for the *endo*-CH of model compound **2**, a small shift for the NH protons is observed. However, for DMSO, the CIS of the NH is larger for the concave host **1** than for model compound **2** (0.57 ppm for **1** and 0.25 ppm for **2**), in contrast to the results of the anion-binding experiments (see above). To the best of our knowledge, this is the second host capable of binding DMSO in an organic solvent [17]. In this regard, concave host **1** seems to be selective and a better binder for negatively polarized oxygen atoms when compared to acyclic compound **2**.

Next, element oxides other than sulfoxides were chosen as guests, namely pyridine-*N*-oxide (PyNO) [18–19] and triphenylphosphine oxide (TPPO). PyNO showed the same behaviour as DMSO, i.e., large CIS for concave host **1**, and small CIS for the linear compound (Figure 2, PyNO, *endo*-CH, 0.34 ppm for **1** and 0.14 ppm for **2**). In contrast, with TPPO as guest, model compound **2** showed a larger CIS when compared to concave host **1** (Figure 2, TPPO). This may be explained by the large steric bulk of TPPO, which may be too extreme to allow TPPO to fit nicely inside the cavity of concave host **1** but still allows a binding to the sterically less demanding non-macrocyclic host **2**.

In order to reliably determine small binding constants, a titration up to a large excess of guest has to be carried out but, even then, a limiting value for the CIS often is not reached and thus a second parameter besides *K*_ass_, namely the maximum of the observed CIS Δδ_max_, has to be obtained by curve fitting, which adds to the overall error. In our host–guest systems, saturation was not reached even when 20 equivalents of guests were used. An even larger excess of guest changed the polarity of the solvent to such an extent that all signals were affected, not only those involved in the binding [20].

If most guests only bind very moderately, an exact (and tedious) determination of all binding constants *K*_ass_ is not interesting. It would be sufficient if a quick screening of the binding potentials of the hosts were possible and host–guest pairs with significant association constants were identified. Estimation rather than an exact determination of *K*_ass_ would be fair enough. Once a good candidate is recognized, a standard determination of the association constant, for example, by NMR titration, can be done.

With Δδ_max_ unknown, the magnitude of the CIS cannot distinguish between weak and strong binding. However, when NMR titrations of different host–guest pairs are carried out with identical concentrations, small and large association constants can be differentiated by the different curvatures of the titration graphs. In a titration curve of strong binding, the curvature is more extreme, and the final value of Δδ_max_ is approached faster than in the case of weak binding. Beyond 1:1 stoichiometry, the CIS values converge more the stronger the binding is.

**Figure 3:**
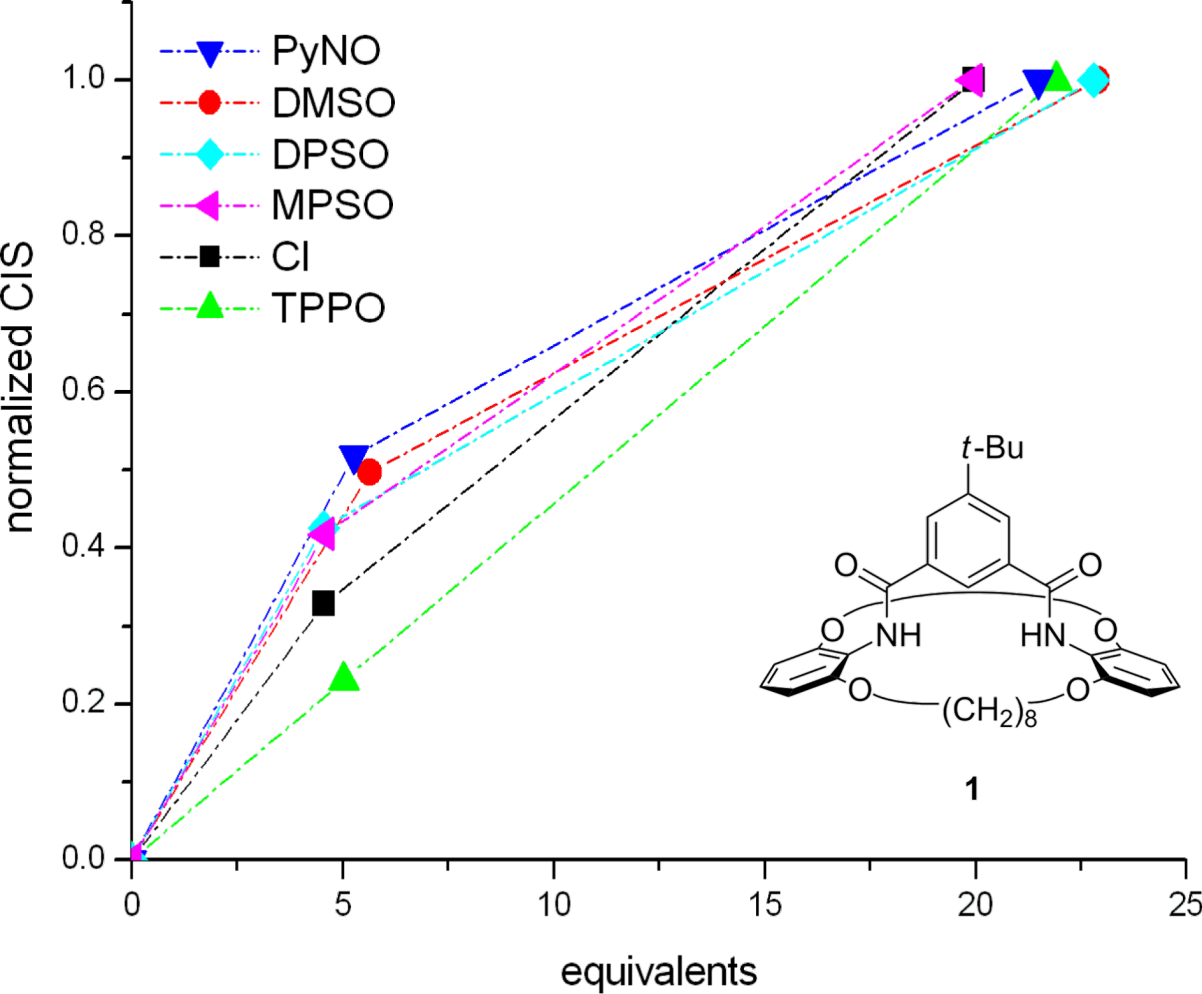
Normalized ^1^H NMR CIS (see text) for concave host **1** with different anionic and neutral guests. The deviations of the data points at ca. 5 equivalents, from the straight line connecting the origin and the data points at 20 equivalents, describe the strength of binding. The binding strength decreases from top to bottom. The dashed lines have no physical significance but help to demonstrate the deviation from linearity.

Can this be a method for the quick estimation of binding constants? We have tested this alternative for hosts **1** and **2**. All measured ^1^H NMR shifts were normalized to a CIS at high guest concentration, but not at saturation: The CIS from all experiments that used ca. 20 equivalents of a given guest were arbitrarily defined as 100%, and the CIS measured for ca. 5 equivalents of the same guest were divided by that CIS value measured with 20 equivalents. The resulting normalized CIS were plotted against the guest equivalents (Figure 3). With a more strongly binding guest, the titration curve possesses a more extreme curvature, and thus, in this normalized form, the data points at 5 equivalents lie further away from the linear line that connects the points corresponding to 0 and 20 equivalents.

**Figure 4:**
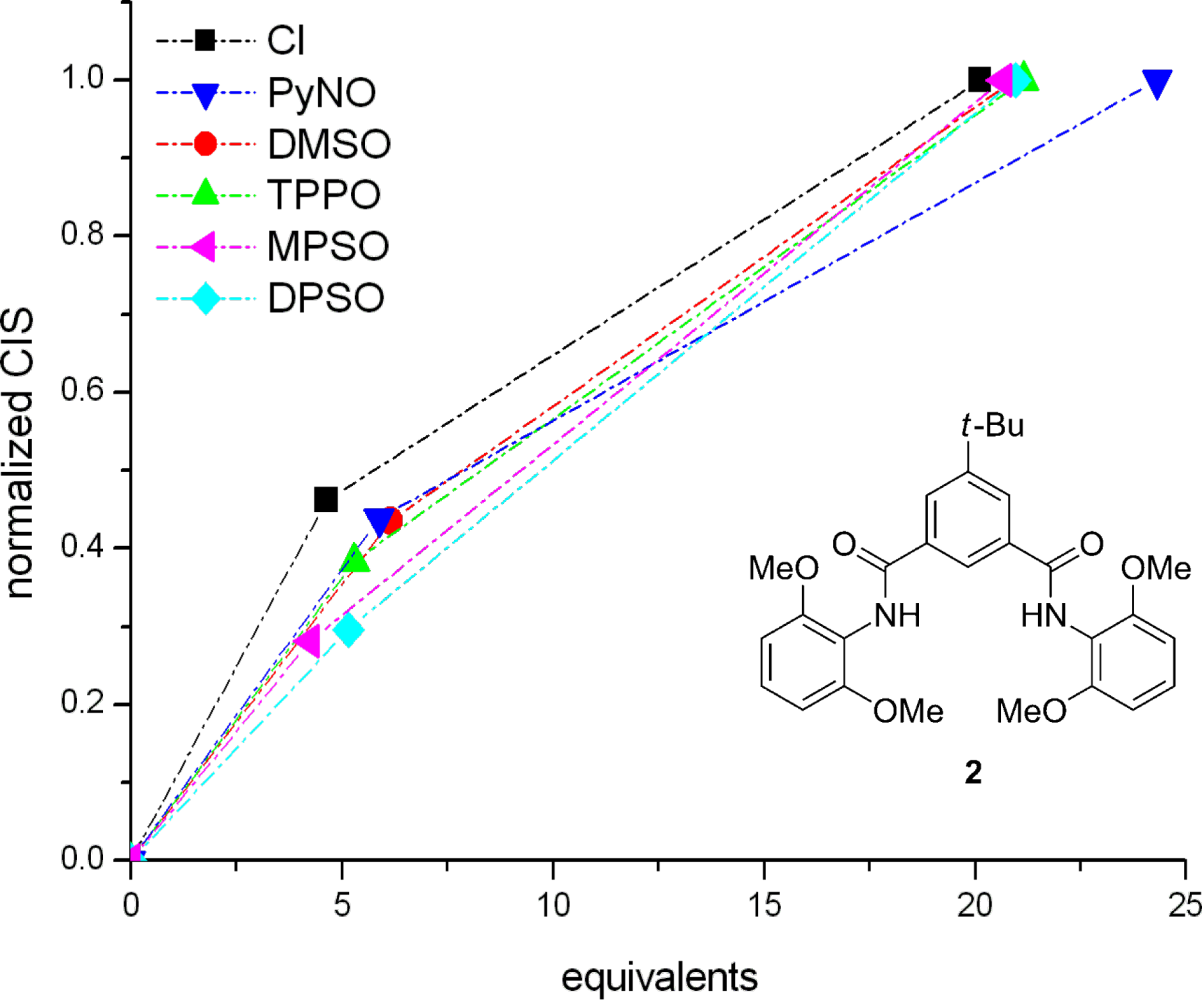
Normalized ^1^H NMR CIS (see text) for concave host **2** with different anionic and neutral guests. The deviations of the data points at ca. 5 equivalents, from the straight line connecting the origin and the data points at 20 equivalents, describe the strength of binding. The binding strength decreases from top to bottom. The dashed lines have no physical significance but help to demonstrate the deviation from linearity.

The validity of this estimation has been checked with calculated titration curves for different association constants *K*_ass_ and different maximum CIS (see Supporting Information File 1). For an application on host **1**, see Figure 3; for **2**, see Figure 4. For each host, only those guests that are bound most strongly are listed. For the full data set, see Supporting Information File 1. Data points below the straight line are physically meaningless, and simply reflect the large errors for very weak binding (the method of normalizing the shifts should preferably not be carried out for guests with *very* small CIS).

In Figures 3 and 4, the relative strengths of binding can be obtained from the vertical deviations of the normalized CIS at ca. 5 equivalents from the straight line connecting the origin and the values at 20 equivalents. The magnitude of the binding decreases from top to bottom.

By using this methodology, it is possible and easy to determine the relative binding strengths of the two hosts **1** and **2** even for weak binding constants and situations where maximal chemically induced shifts Δδ_max_ cannot be determined from only a few measurements. When the two graphs for **1** and **2** with different guests are compared, the different selectivity of the two hosts becomes evident. Concave host **1** shows a better affinity for negatively polarized oxygen atoms than for anions, except in the case of the bulky TPPO. The affinities of concave host **1** lie in the following order: PyNO > DMSO > DPSO = MPSO > Cl^−^ > TPPO. In contrast, the affinities of the non-macrocyclic analogue **2** are: Cl^−^ > PyNO = DMSO = TPPO > MPSO = DPSO (see Supporting Information File 1). When the plot was compared with the simulated titration curves (see Supporting Information File 1, page S12), *K*_ass_ for the best binder to **1**, pyridine-*N*-oxide (PyNO), was estimated to be ca. 40 M^−1^. A subsequent NMR titration of host **1** with PyNO provided an association constant of 25 M^−1^ (see Supporting Information File 1). Remarkably, chloride ions are only very weakly bound by concave host **1**, and binding constants are moderate anyway. A possible explanation may be a repulsion between the negatively polarized oxygen atoms in the 2- and 6-positions of the bridge heads of **1** and the negatively, or partially negatively charged guests.

## Conclusion

**1** is a readily synthesized concave host molecule in which the isophthalamide moiety is the central binding unit, and it is preorganized by its incorporation into the bi-macrocyclic structure. This concave host, although it does not exhibit strong binding, is selective for negatively polarized oxygen atoms and selects them according to the steric bulk of the guests. These initial experiments now open the way for the synthesis of new modified concave hosts based on isophthalamide units with improved binding selectivity and/or for organocatalysis [21]. Concave host **1** can also be applied as a carrier in transport experiments. When applied to chloride-loaded liposomes [22], it showed twice as much transmembrane chloride transport with respect to acyclic compound **2** (see Supporting Information File 1). Even if the chloride binding is lower for concave host **1**, the transport through a bilayer membrane is faster. Additional transport experiments are under investigation.

## Experimental

### General remarks

All reagents were obtained from commercial sources and used without additional purification unless otherwise indicated. 5-*tert*-Butylisophthaloyl dichloride (**3**) was prepared from 5-*tert*-butylisophthalic acid and thionyl chloride according to Vögtle et al. [23]. 2,6-Bis(pent-4-enyloxy)aniline (**4**) was prepared from 2-nitroresorcine according to Winkelmann et al. [16]. 2,6-Dimethoxyaniline (**6**) was synthesized from 2-nitroresorcine according to Mechoulam and Srebnik [24] and was finally reduced to 2,6-dimethoxyaniline (**6**) according to Franck and Kauffmann [25]. THF was freshly distilled from lithium aluminium hydride (triphenylmethane as indicator). All reactions were carried out in an atmosphere of nitrogen. NMR spectra were recorded with Bruker AC 200, DRX 500 or AV 600 instruments. Assignments are supported by COSY, HSQC and HMBC. All chemical shifts are referenced to TMS or residual solvent peaks. Mass spectra were recorded with Finnigan MAT 8200 or MAT 8230. ESI mass spectra were recorded with an Applied Biosystems Mariner Spectrometry Workstation. IR spectra were recorded with Perkin-Elmer Spectrum 100 spectrometer, equipped with an ATR unit. Elemental analyses were carried out with a EuroEA 3000 Elemental Analyzer from Euro Vector. MALDI-TOF spectra were recorded with Bruker-Daltonics Biflex III. 4-Chloro-α-cyanocinnamic acid (Cl-CCA) was used as the matrix.

### ^1^H NMR experiments

Each NMR tube was filled with 600 µL of a stock solution (5 mg/mL) of **1** or **2** in CD_2_Cl_2_, and subsequently ca. 5, and later ca. 20 equivalents of the respective guest were added. The exact amount was recalculated from the integrals by using Bruker Topspin^®^ 2.1. All experiments were carried out on a Bruker AC 200 NMR equipped with an autosampler at 300 K. The spectra are referenced to the residual solvent peak.

#### Synthesis of 25^5^-*tert*-Butyl-2,11,13,22-tetraoxa-23,27-diaza-1,12 (1,3,2)-25 (1,3)-tribenzenabicyclo[10.10.5]heptacosaphan-24,26-dione (**1**)

A solution of 25^5^-*tert*-Butyl-2,11,13,22-tetraoxa-23,27-diaza-1,12(1,3,2)-25(1,3)-tribenzenabicyclo[10.10.5]heptacosaphan-6,17-dien-24,26-dione (186 mg, 284 µmol), Pd/C (10%, 150 mg) and methanol (30 mL) was stirred under an atmosphere of hydrogen for 24 h. The mixture was filtered through a syringe filter (0.450 µm) to remove all Pd/C, and the solvent was removed under reduced pressure to obtain **1** as a white solid (144 mg, 219 µmol, 77%); mp 225 °C (decomp.); ^1^H NMR (500 MHz, CDCl_3_) δ 8.30 (s, 2H, 25^4,6^-H), 8.06 (s, 1H, 25^2^-H), 7.24 (br. s, 2H, NH), 7.14 (t, ^3^*J* = 8.4 Hz, 2H, 1^5^,12^5^-H), 6.59 (d, ^3^*J* = 8.4 Hz, 4H, 1^4,6^,12^4,6^-H), 4.19 (ddd, ^2^*J =* ca. 9 Hz*, *^3^*J* = ca. 9 Hz, ^3^*J* = ca. 2 Hz, 4H, OC*H_a_*H_b_), 3.85 (ddd, ^2^*J* = 9.7 Hz*, *^3^*J* = 9.7 Hz, ^3^*J* = 2.1 Hz, 4H, OCH_a_*H_b_*), 1.79 (m_c_, 4H, OCH_2_C*H_a_*H_b_), 1.62 (m_c_, 4H, OCH_2_CH_a_*H_b_*), 1.43 (s, 9H, CH_3_), 1.32 (m_c_, 8H, CH_2_), 1.25 (m_c_, 8H, CH_2_); ^13^C NMR (125 MHz, CDCl_3_) δ 165.4 (s, *C*=O), 154.0 (s, 1^1,3^,12^1,3^*-*C), 153.3 (s, 25^5^-C), 134.8 (s, 25^1,3^-C), 129.6 (d, 25^4,6^-C), 127.1 (d, 1^5^,12^5^-C), 121.1 (d, 25^2^-C), 115.6 (s, 1^2^,12^2^-C), 105.4 (d, 1^4,6^, 12^4,6^-C), 69.2 (t, OCH_2_), 35.3 (s, *C*(CH_3_)_3_), 31.2 (q, CH_3_), 30.6 (t, OCH_2_*C*H_2_), 29.7 (t, O(CH_2_)_3_*C*H_2_), 27.4 (t, O(CH_2_)_2_*C*H_2_); IR (ATR) 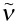: 3430 (w, NH), 2930, 2848 (2 w, aliph. CH), 1683 (m, C=O), 1589 (w, arom. C=C), 1509 (m, arom. C=C),1392 (s, CH_3_) cm^−1^. EIMS (70 eV): *m/z* (% relative intensity) 656 (100) [M]^+•^; CIMS (isobutane): *m/z* (% relative intensity) 657 (30) [M + H]^+^; ESIMS (CHCl_3_): *m/z* (% relative intensity) 679 (100) [M + Na]^+^, 657 (75) [M + H]^+^; MS (MALDI-TOF, Cl-CCA): *m/z* 695 [M + K]^+^, 679 [M + Na]^+^, 656 [M]^+^; HRMS calcd for C_40_H_52_N_2_O_6_ 656.38251; found: 656.38257 (Δ = −0.1 ppm); calcd for C_39_^13^CH_52_N_2_O_6_ 657.38586; found: 657.38597 (Δ = −0.2 ppm); Anal. calcd for C_40_H_52_N_2_O_6_: C, 73.14; H, 7.98; N, 4.26; found: C, 73.04; H, 8.04; N, 4.39.

#### Synthesis of 5-*tert*-Butyl-*N,N'*-bis(2,6-dimethoxyphenyl)-isophthalamide (**2**)

A solution of 5-*tert*-butylisophthaloyl dichloride (**3**, 550 mg, 2.12 mmol) in tetrahydrofuran (5.00 mL) was added dropwise over 45 min to a stirred solution of 2,6-dimethoxyaniline (**6**) (650 mg, 4.24 mmol) and triethylamine (2.35 mL, 1.72 g, 17.0 mmol) in tetrahydrofuran (20 mL). The solution was stirred for 24 h. The solvent and excess of triethylamine was evaporated under reduced pressure. The residue was dissolved in chloroform (25 mL) and water (25 mL). The water phase was extracted once with chloroform (25 mL), the combined organic layer was dried with magnesium sulfate and evaporated under reduced pressure to yield a yellow solid, which was purified by column chromatography (silica gel, dichloromethane/methanol, 40:1, *R*_f_ = 0.11) to give **2** as a white solid (1.02 g, 2.07 mmol, 97%); mp 122 °C; ^1^H NMR (500 MHz, CDCl_3_) δ 8.24 (s, 1H, Ar^1^-2-H), 8.14 (s, 2H, Ar^1^-4,6-H), 7.43 (br. s, 2H, NH), 7.21 (t, ^3^*J* = 8.4 Hz, 2H, Ar^2^-4-H), 6.62 (d, ^3^*J* = 8.4 Hz, 4H, Ar^2^-3,5-H), 3.84 (s, 12H, OCH_3_), 1.38 (s, 9H, CH_3_); ^13^C NMR (125 MHz, CDCl_3_) δ 165.8 (C=O), 155.1 (Ar^2^-2,6-C), 152.4 (Ar^1^-5-C), 134.7 (Ar^1^-1,3-C), 128.2 (Ar^1^-4,6-C), 127.6 (Ar^2^-4-C), 123.4 (Ar^1^-2-C), 114.6 (Ar^2^-1-C), 104.4 (Ar^2^-3,5-C), 56.0 (OCH_3_), 35.1 (*C*(CH_3_)_3_), 31.3 (q, CH_3_); IR (ATR) 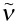: 3387 (w, NH), 3231 (w, arom. CH), 2947 (m, aliph. CH), 1663 (m, C=O), 1593 (m, arom. C=C), 1519 (s, arom. C=C) cm^−1^; EIMS (70 eV): *m/z* (% relative intensity) 492 (83) [M]^+•^, 340 (100) [M − C_8_H_10_NO_2_]^+^; CIMS (isobutane): *m/z* (% relative intensity) 493 (100) [M + H]^+^. Anal. calcd for C_28_H_32_N_2_O_6_·0.1CH_2_Cl_2_: C, 67.36; H, 6.48; N, 5.59; found: C, 67.42; H, 6.53; N, 5.74.

#### Synthesis of *N,N'*-Bis(2,6-bis[pent-4-enyloxy]-phenyl)-5-*tert*-butyl-isophthalamide (**5**)

In 20 mL anhydrous tetrahydrofuran, 2,6-bis(pent-4-enyloxy)aniline (4, 1.95 g, 7.46 mmol) and anhydrous triethylamine (4.14 mL, 3.02 g, 29.7 mmol) were dissolved. A solution of 5-*tert*-butylisophthaloyl dichloride (**3**) (970 mg, 3.75 mmol) in anhydrous tetrahydrofuran (10 mL) was added dropwise. The solution was stirred for 16 h at room temperature. The solvent and excess of triethylamine were evaporated under reduced pressure and the residue was dissolved in chloroform (25 mL) and water (25 mL). The aqueous phase was extracted with chloroform (25 mL). The organic layers were collected, dried with magnesium sulfate and the solvent was evaporated under reduced pressure. The product was isolated by column chromatography (silica, cyclohexane/ethyl acetate, 1:1, *R*_f_ = 0.34) as a white solid (1.63 g, 2.30 mmol, 61%); mp 136 °C; ^1^H NMR (500 MHz, CDCl_3_) δ 8.18 (s, 1H, Ar^1^-2-H), 8.08 (s, 2H, Ar^1^-4,6-H), 7.28 (br. s, 2H, NH), 7.16 (t, ^3^*J* = 8.4 Hz, 2H, Ar^2^-4-H), 6.59 (d, ^3^*J* = 8.4 Hz, 4H, Ar^2^-3,5-H), 5.78 (ddt, ^3^*J* = 16.9 Hz, ^3^*J* = 10.2 Hz, ^3^*J* = 6.6 Hz, 4H, C*H*=CH_2_), 4.97 (m_c_, 4H, H_Z_), 4.91 (m_c_, 4H, H_E_), 4.01 (t, ^3^*J* = 6.5 Hz, 8H, OCH_2_), 2.16 (m_c_, 8H, C*H*_2_CH=CH_2_), 1.85 (m_c_, 8H, OCH_2_C*H*_2_), 1.38 (s, 9 H, CH_3_).^13^C NMR (125 MHz, CDCl_3_) δ 166.1 (s, C=O), 154.8 (s, Ar^2^-2,6-C), 152.3 (s, Ar^1^-5-C), 137.6 (d, *C*H=CH_2_), 135.4 (s, Ar^1^-1,3-C), 127.6 (d, Ar^1^-4,6-C), 127.6 (d, Ar^2^-4-C) 123.3 (d, Ar^1^-2-C), 115.1 (t, CH=*C*H_2_), 106.2 (s, Ar^2^-1-C), 105.4 (d, Ar^2^-3,5-C), 68.1 (t, OCH_2_), 35.1 (s, *C*(CH_3_)_3_), 31.2 (q, CH_3_), 30.1 (t, *C*H_2_CH=CH_2_), 28.4 (t, OCH_2_*C*H_2_); IR (ATR) 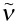: 3207 (br. w, NH), 3076 (w, arom. CH), 2946 (m, aliph. CH), 1669 (m, C=O), 1647 (m, aliph. C=C), 1589 (m, arom. C=C), 1520 (s, arom. C=C) cm^−1^; EIMS (70 eV): *m/z* (% relative intensity) 709 (49) [M]^+•^, 708 (100) [M − H]^+^; CIMS (isobutane): *m/z* (% relative intensity) 710 (25) [M + H]^+^, 709 (59) [M]^+•^, 708 (100) [M − H]^+^; ESIMS (CHCl_3_): *m/z* (% relative intensity) 732 (25) [M + Na]^+^; HRMS: calcd for C_44_H_56_N_2_O_6_: 708.41382; found: 708.41390 (Δ = −0.1 ppm); calcd for C_43_^13^CH_56_N_2_O_6_: 709.41718; found: 709.41706 (Δ = 0.2 ppm). Anal. calcd for C_44_H_56_N_2_O_6_·0.3C_6_H_12_·0.3C_4_H_8_O_2_: C, 74.18; H, 8.24; N, 3.66; found: C, 73.95; H, 7.93; N, 4.04.

#### Synthesis of 25^5^-*tert*-Butyl-2,11,13,22-tetraoxa-23,27-diaza-1,12(1,3,2)-25(1,3)-tribenzenabicyclo[10.10.5]heptacosaphan-6,17-dien-24,26-dione

Anhydrous dichloromethane (800 mL) was added to a mixture of *N,N'*-bis-(2,6-bis[pent-4-enyloxy]-phenyl)-5-*tert*-butyl-isophthalamide (**5**, 1.00 g, 1.41 mmol) and Grubbs Catalyst 1st gen. (162 mg, 141 µmol). The solution was stirred for 24 h at room temperature. The reaction was quenched with ethyl vinyl ether (2 mL) and the mixture was stirred for 1 h. The solvent was removed under reduced pressure and the crude product was filtered over silica gel (1 cm, dichloromethane/methanol, 40:1). The solvent was removed and cyclohexane/ethyl acetate (150 mL, 1:1, v/v) was added to crystallize the product. The product was filtered off and washed with ethyl acetate (10 mL) to obtain a white solid (186 mg, 284 µmol, 20%). ^1^H NMR (500 MHz, CDCl_3_) δ 8.25 (s, 2H, 25^4,6^-H), 7.98 (s, 1H, 25^2^-H), 7.20 (br. s, 2H, NH), 7.15 (t, *^3^J* = 8.3 Hz, 2H, 1^5^,12^5^-H), 6.61 (d, ^3^*J* = 8.3 Hz, 4H, 1^4,6^,12^4,6^-H), 5.36–5.27 (m, 4H, C*H*=C*H*), 4.22–3.82 (m, 8H, OCH_2_), 2.20–1.60 (m, 16H, CH_2_), 1.42 (s, 9H, C*H_3_*) ppm; ^13^C NMR (125 MHz, CDCl_3_) δ 154.2 (s, 1^1,3^,12^1,3^*-*C), 153.8 (s, 25^5^-C), 134.6 (s, 25^1,3^-C), 130.3 (s, *C*H=*C*H), 129.6 (d, 25^4,6^-C), 127.0 (d, 1^5^,12^5^-C), 121.7 (d, 25^2^-C), 116.1 (s, 1^2^,12^2^-C), 105.4 (d, 1^4,6^,12^4,6^-C), 69.5 (t, OCH_2_), 35.3 (s, *C*(CH_3_)_3_), 31.2 (q, CH_3_), 30.7 (t, OCH_2_*C*H_2_), 24.9 (t, O(CH_2_)_2_*C*H_2_); The C=O signal was too weak to be detected in this ^13^C spectrum. MS (MALDI-TOF, Cl-CCA): *m/z* 676 [M + Na]^+^, 654 [M + H]^+^.

## Supporting Information

NMR spectra and product analyses for **1** and **2** are available in the Supporting Information as well as details of the NMR CIS titrations, the evaluation of the normalized CIS method, ^1^H,^1^H NOESY experiments, and the transport experiments.

File 1 Product analyses and experimental data.
