# Supplementary material for: Binding of group 15 and group 16 oxides by a concave host containing an isophthalamide unit
Source: Beilstein J Org Chem. 2012 Jan 3;8:11–7. doi: 10.3762/bjoc.8.2 (PMC3302070; doi:10.3762/bjoc.8.2)
Supplement: File 1 — Product analyses and experimental data. [file Beilstein_J_Org_Chem-08-11-s001.pdf]

Supporting Information

for

Binding of group 15 and group 16 oxides by a concave host containing an isophthalamide unit

Jens Eckelmann, Vittorio Saggiomo, Svenja Fischmann and Ulrich Lüning*

Address: [1]Otto-Diels-Institut für Organische Chemie, Christian-Albrechts-Universität zu Kiel, Olshausenstr. 40, D-24098 Kiel, Germany

Email: Ulrich Lüning* - luening@oc.uni-kiel.de

*Corresponding author

Product analyses and experimental data

Contents

25^5-*tert*-Butyl-2,11,13,22-tetraoxa-23,27-diaza-1,12(1,3,2)-25(1,3)-tribenzenabicyclo[10.10.5]heptacosaphan-24,26-dione (**1**)

^{1}H NMR S2

^{13}C NMR S3

5-*tert*-Butyl-*N,N'*-bis(2,6-dimethoxyphenyl)-isophthalamide (**2**)

^{1}H NMR S4

^{13}C NMR S5

^{1}H NMR experiments with different guests S6

Normalized CIS of **1** S9

Normalized CIS of **2** S10

Evaluation of the Normalized CIS Method S11

^{1}H NMR titration of **1** and pyridine-*N*-oxide S17

^{1}H,^{1}H NOESY experiments S18

Transport experiments S20

References S23

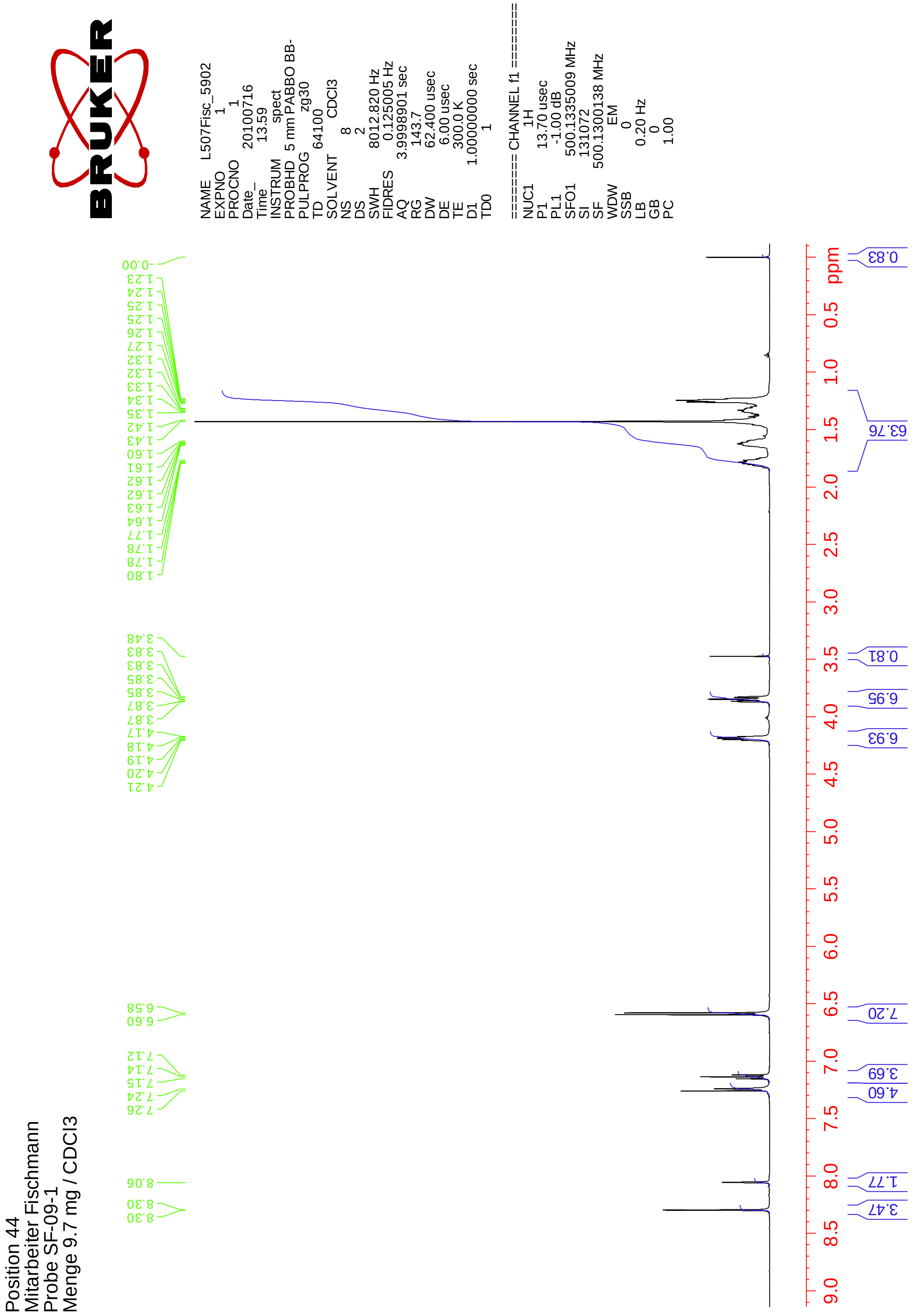

Figure S1: ^{1}H NMR (500 MHz, 300 K, $CDCl_3$) of **1.**

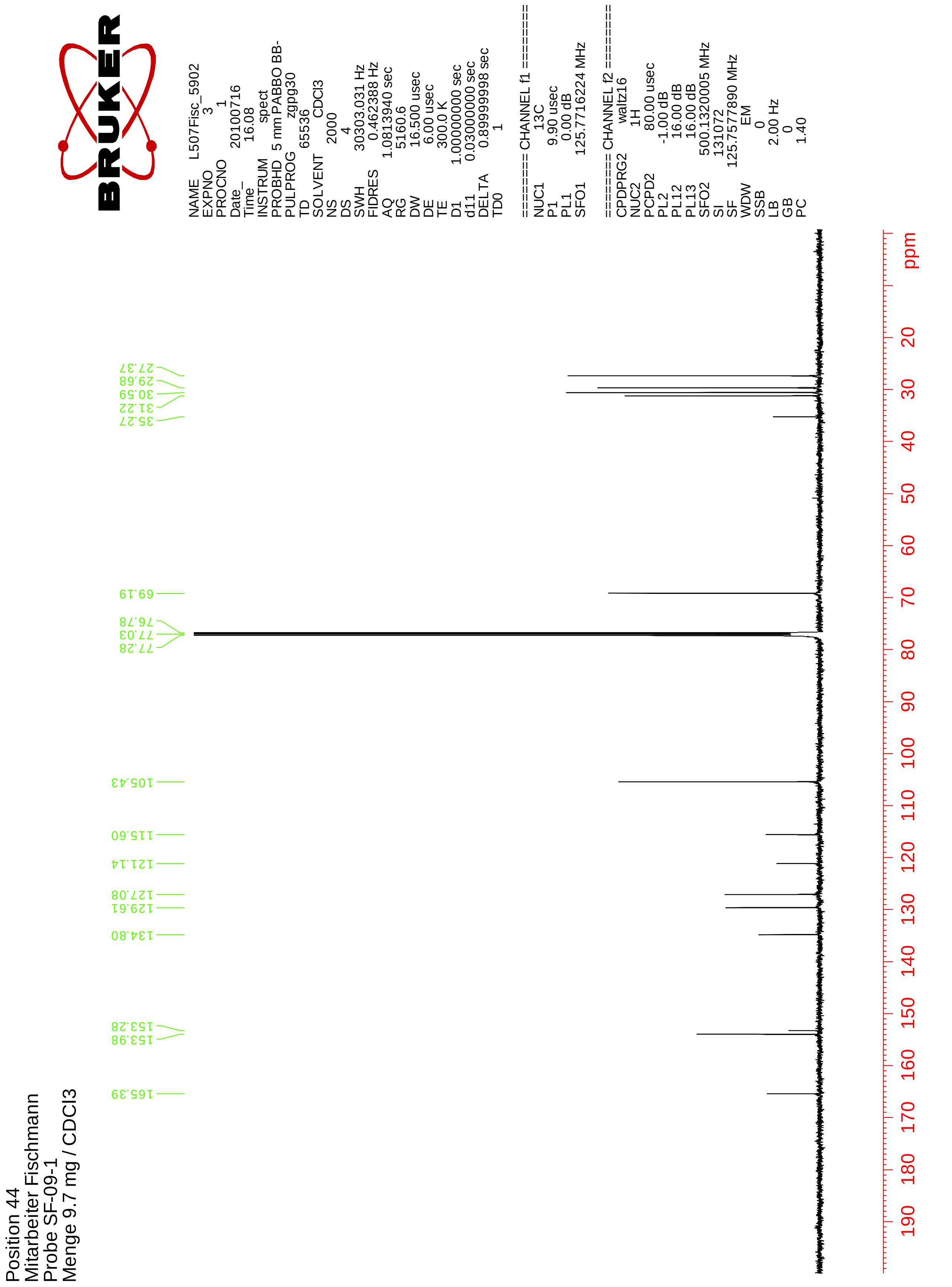

Figure S2: ^{13}C NMR (125 MHz, 300 K, $CDCl_3$) of **1**.

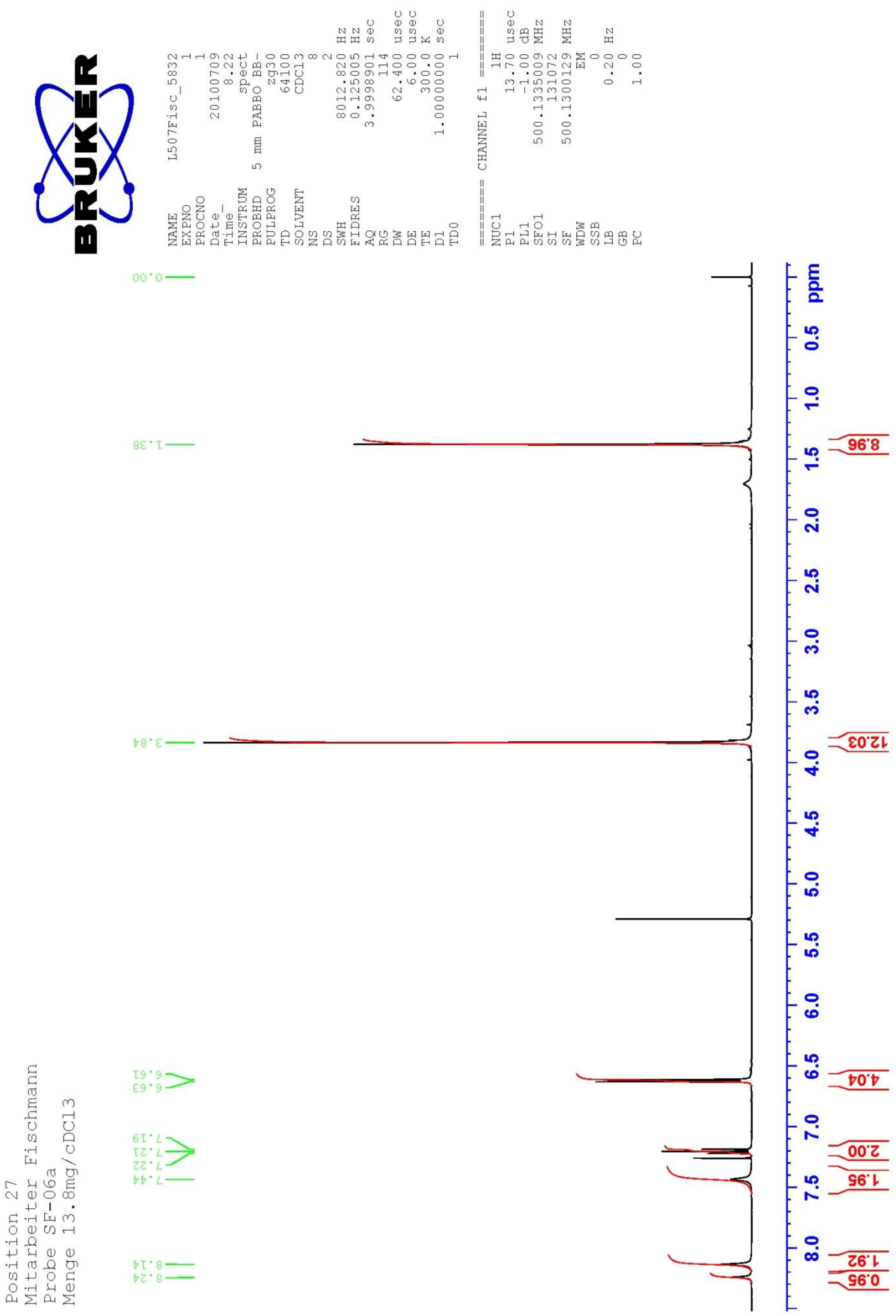

Figure S3: ^{1}H NMR (500 MHz, 300 K, $CDCl_3$) of **2**.

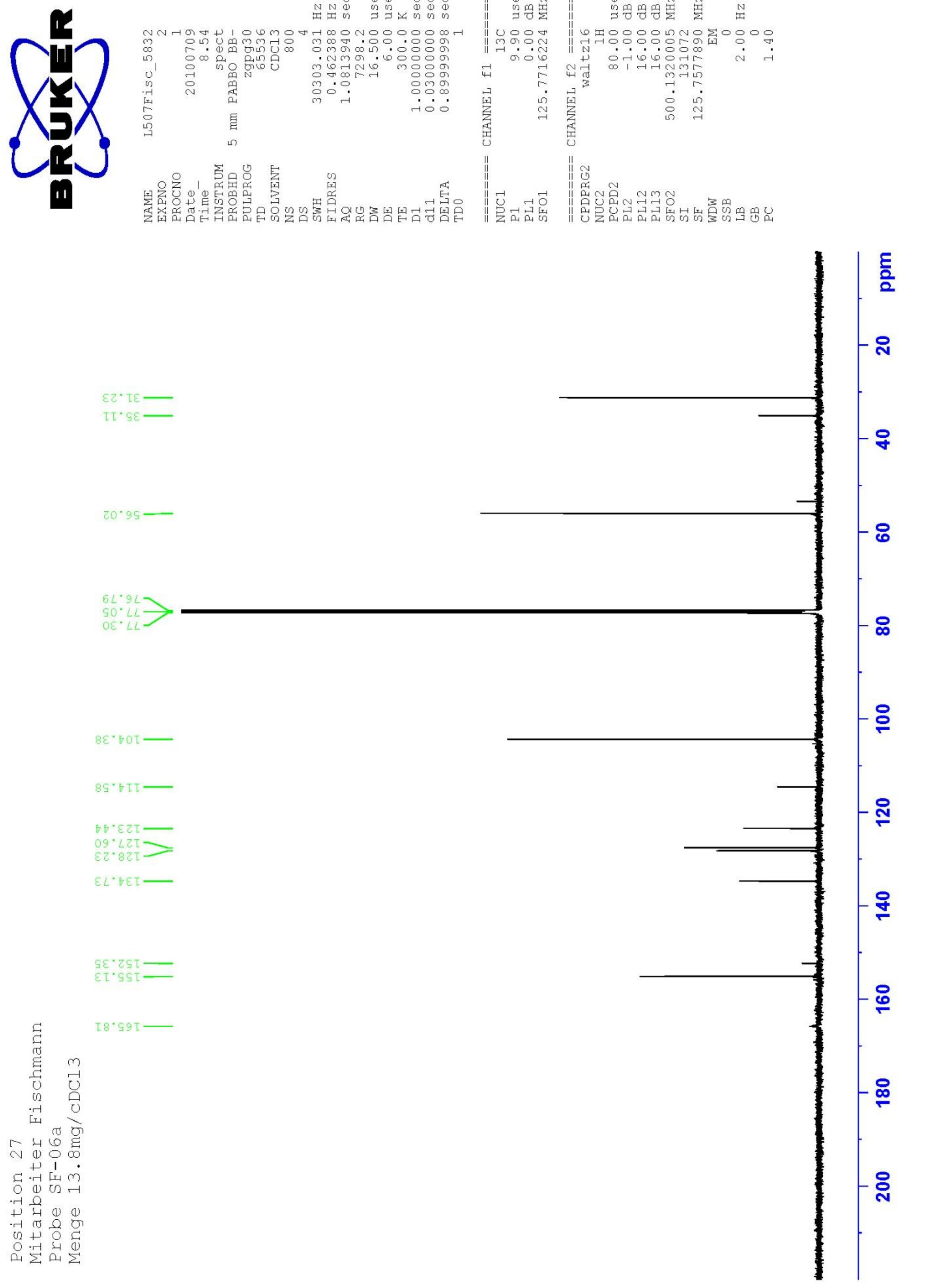

Figure S4: ^{13}C NMR (125 MHz, 300 K, $CDCl_3$) of **2**.

NMR-Experiments

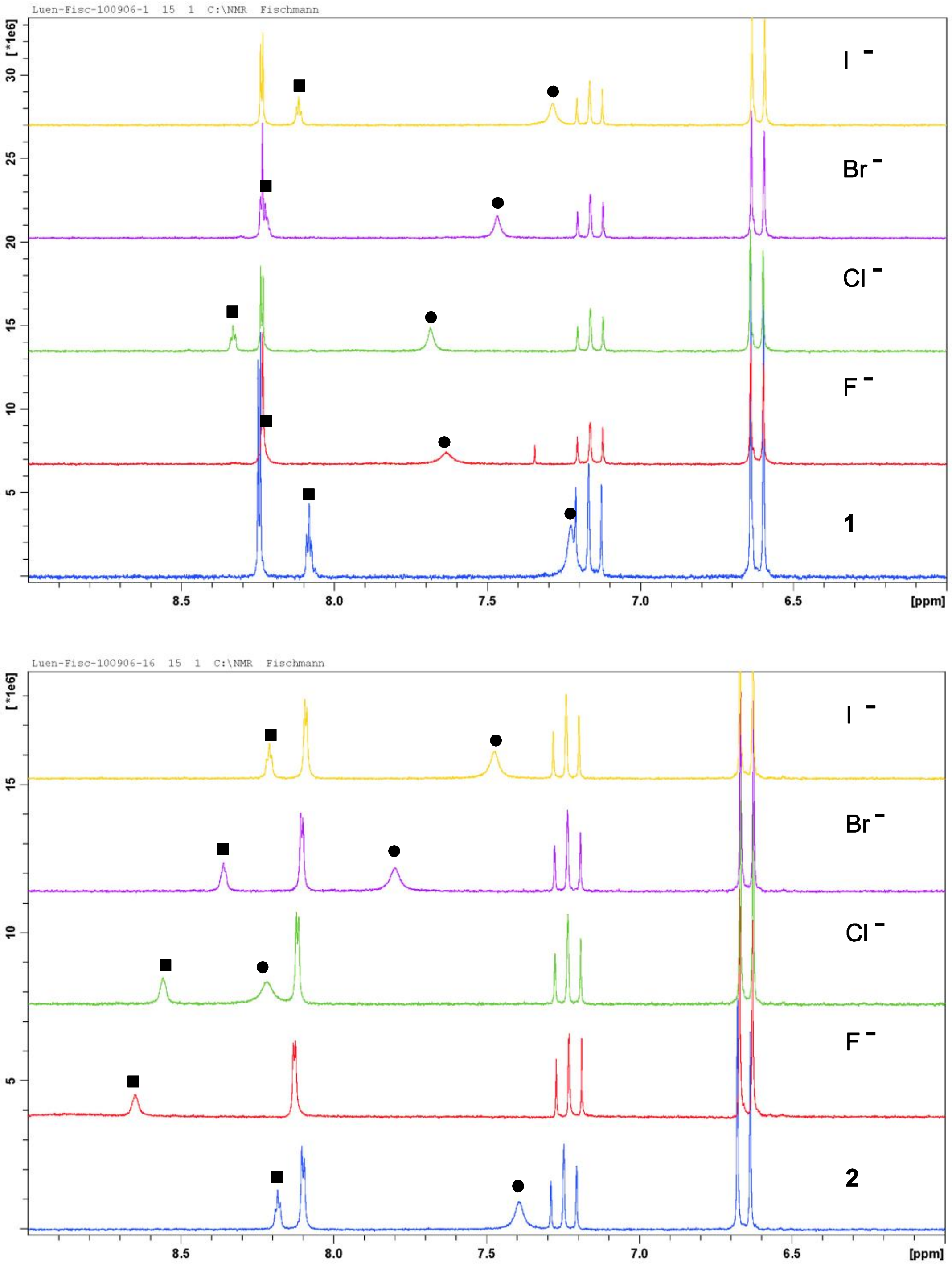

Figure S5: Binding studies with different tetrabutylammonium halides (5 equivalents; top: bi-macrocycle **1**, bottom: isophthalamide **2**). The guests were added in solid form and the exact amount was calculated from the ^{1}H NMR integration.

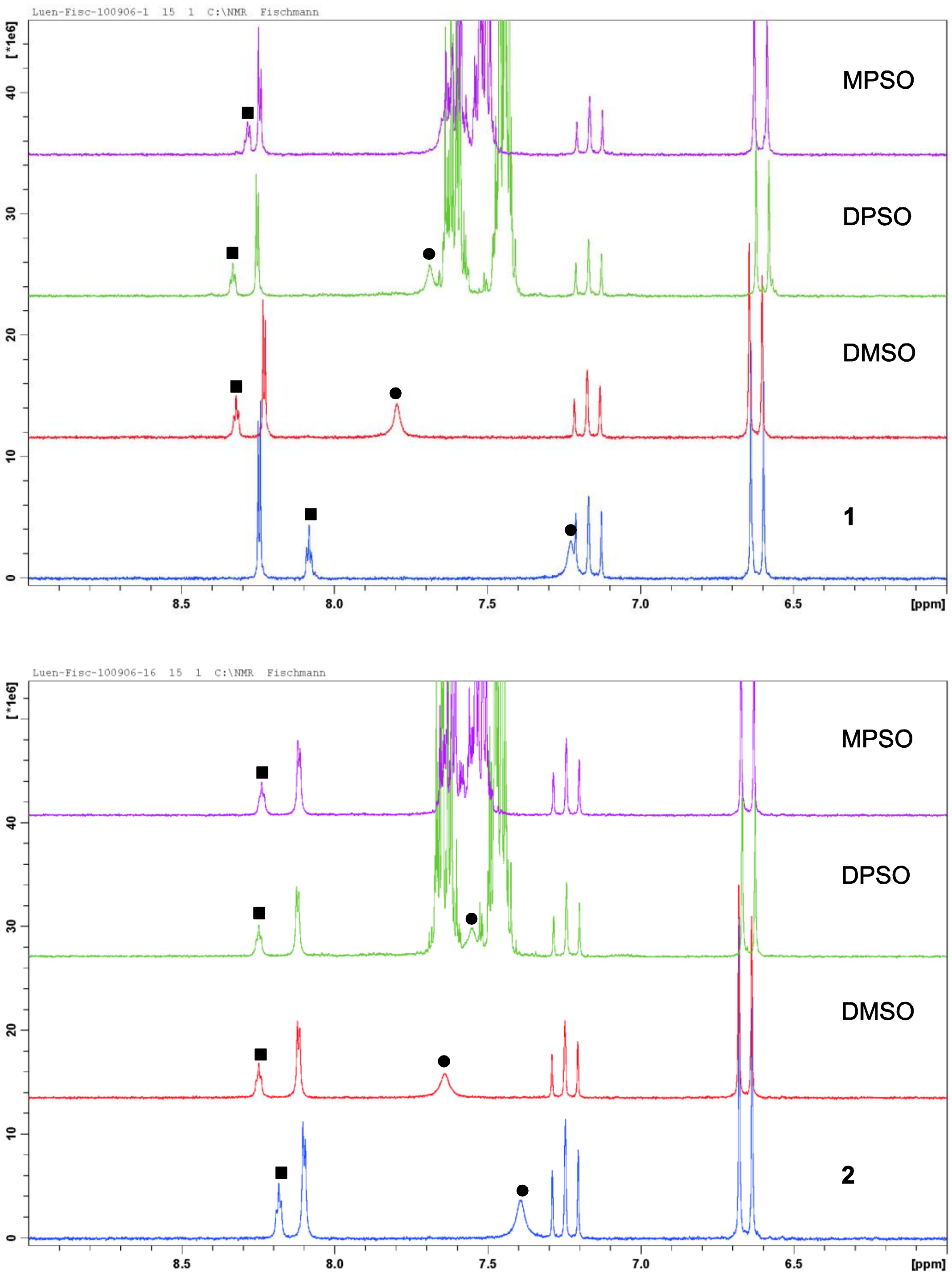

Figure S6: Binding studies with different sulfoxides (5 equivalents; top: bi-macrocycle **1**, bottom: isophthalamide **2**, MPSO = methylphenyl sulfoxide, DPSO = diphenyl sulfoxide, DMSO = dimethyl sulfoxide). The guests were added undiluted and the exact amount was calculated from the ^{1}H NMR integration.

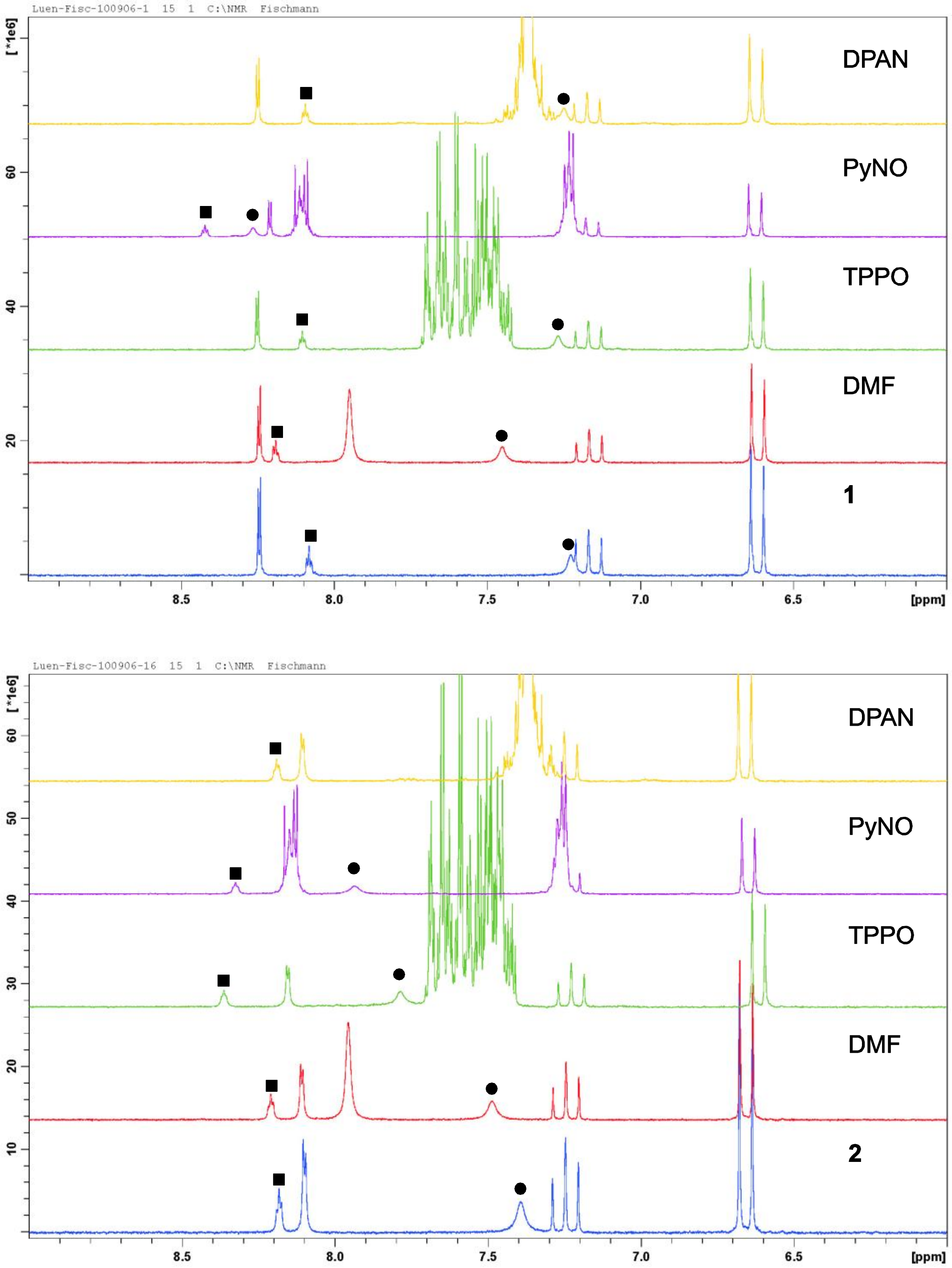

Figure S7: Binding studies with different guests (5 equivalents; top: bi-macrocycle **1**, bottom: isophthalamide **2**, DPAN = diphenylacetonitrile, PyNO = pyridine-*N*-oxide, TPPO = triphenylphosphine oxide, DMF = *N,N*-dimethylformamide). The guests were added undiluted and the exact amount was calculated from the ^{1}H NMR integration.

Normalized CIS of 1

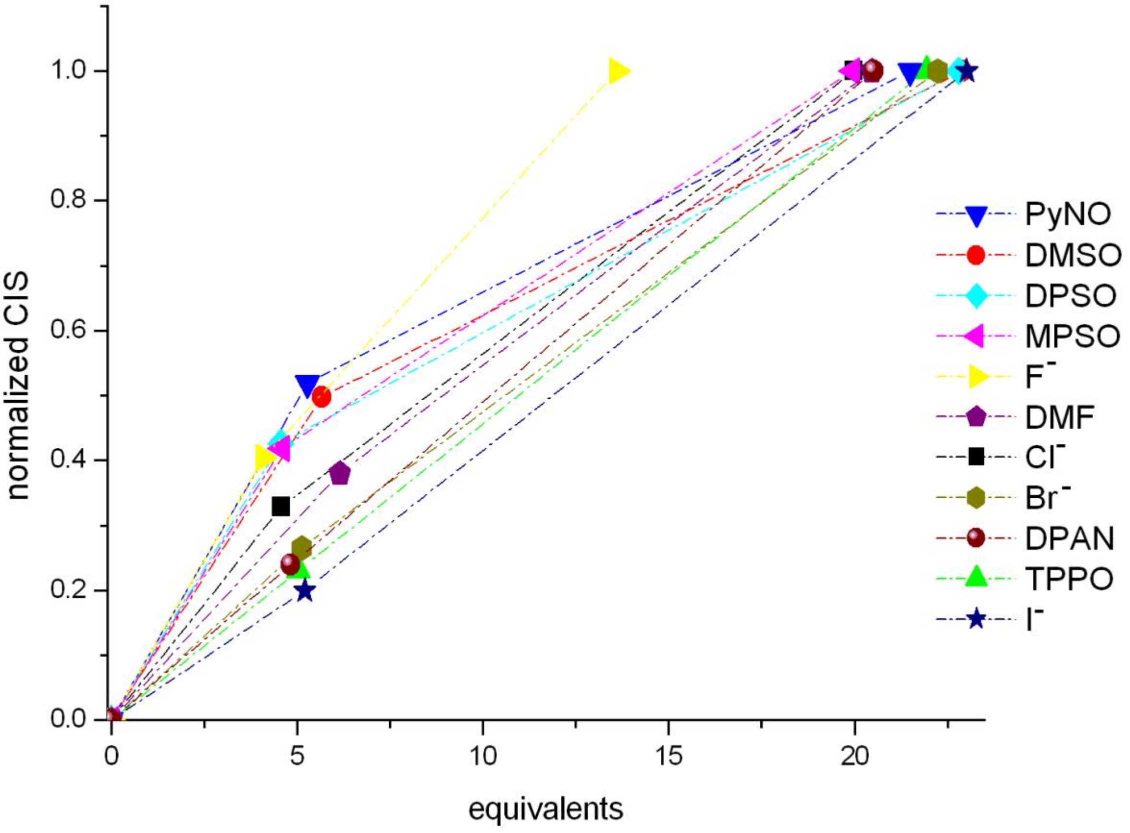

Figure S8: Normalized CIS of **1** (isophthalamide *endo*-CH proton).

Normalized CIS of 2

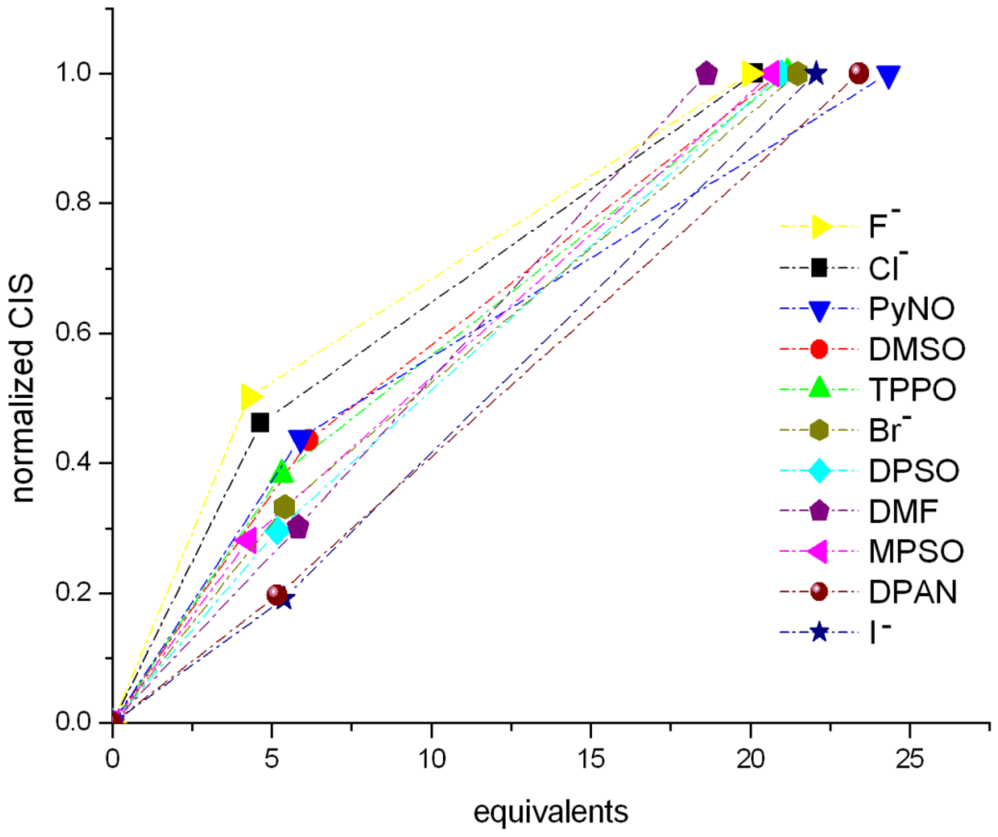

Figure S9: Normalized CIS of **2** (isophthalamide *endo* CH proton).

Evaluation of the Normalized CIS Method

To prove that normalized CIS is a valid method to estimate the binding strength, six different titration curves were simulated. Association constants between K_{ass} = 2 and K_{ass} = 100 were chosen. Host concentration was fixed at
0.003 mol L^{-1}.
Equations used:

$H := 0.003$

$G := 0, 0.0001 .. 0.06$

$\Delta\delta_{max} := 1$

K = association constant (M^{-1})
G = concentration guest (M)
H = concentration host (M)
$\Delta\delta_{max}$ =shift(G_{sat}) - shift (G_0) (ppm)

$K := 2$

$$K_2(G) := \left[\left[\frac{\Delta\delta_{max}}{(2\cdot H)}\right]\cdot\left[\left[G + H + \left(\frac{1}{K}\right)\right] - \left[\left[(G - H)^2\right] + \left(2\cdot\frac{G}{K}\right) + \left(2\cdot\frac{H}{K}\right) + \left[\frac{1}{(K)^2}\right]\right]^{0.5}\right]\right]$$

$K := 5$

$$K_5(G) := \left[\left[\frac{\Delta\delta_{max}}{(2\cdot H)}\right]\cdot\left[\left[G + H + \left(\frac{1}{K}\right)\right] - \left[\left[(G - H)^2\right] + \left(2\cdot\frac{G}{K}\right) + \left(2\cdot\frac{H}{K}\right) + \left[\frac{1}{(K)^2}\right]\right]^{0.5}\right]\right]$$

$K := 10$

$$K_{10}(G) := \left[\left[\frac{\Delta\delta_{max}}{(2\cdot H)}\right]\cdot\left[\left[G + H + \left(\frac{1}{K}\right)\right] - \left[\left[(G - H)^2\right] + \left(2\cdot\frac{G}{K}\right) + \left(2\cdot\frac{H}{K}\right) + \left[\frac{1}{(K)^2}\right]\right]^{0.5}\right]\right]$$

$K := 20$

$$K_{20}(G) := \left[\left[\frac{\Delta\delta_{max}}{(2\cdot H)}\right]\cdot\left[\left[G + H + \left(\frac{1}{K}\right)\right] - \left[\left[(G - H)^2\right] + \left(2\cdot\frac{G}{K}\right) + \left(2\cdot\frac{H}{K}\right) + \left[\frac{1}{(K)^2}\right]\right]^{0.5}\right]\right]$$

$K := 50$

$$K_{50}(G) := \left[\left[\frac{\Delta\delta_{max}}{(2\cdot H)}\right]\cdot\left[\left[G + H + \left(\frac{1}{K}\right)\right] - \left[\left[(G - H)^2\right] + \left(2\cdot\frac{G}{K}\right) + \left(2\cdot\frac{H}{K}\right) + \left[\frac{1}{(K)^2}\right]\right]^{0.5}\right]\right]$$

$K := 100$

$$K_{100}(G) := \left[\left[\frac{\Delta\delta_{max}}{(2\cdot H)}\right]\cdot\left[\left[G + H + \left(\frac{1}{K}\right)\right] - \left[\left[(G - H)^2\right] + \left(2\cdot\frac{G}{K}\right) + \left(2\cdot\frac{H}{K}\right) + \left[\frac{1}{(K)^2}\right]\right]^{0.5}\right]\right]$$

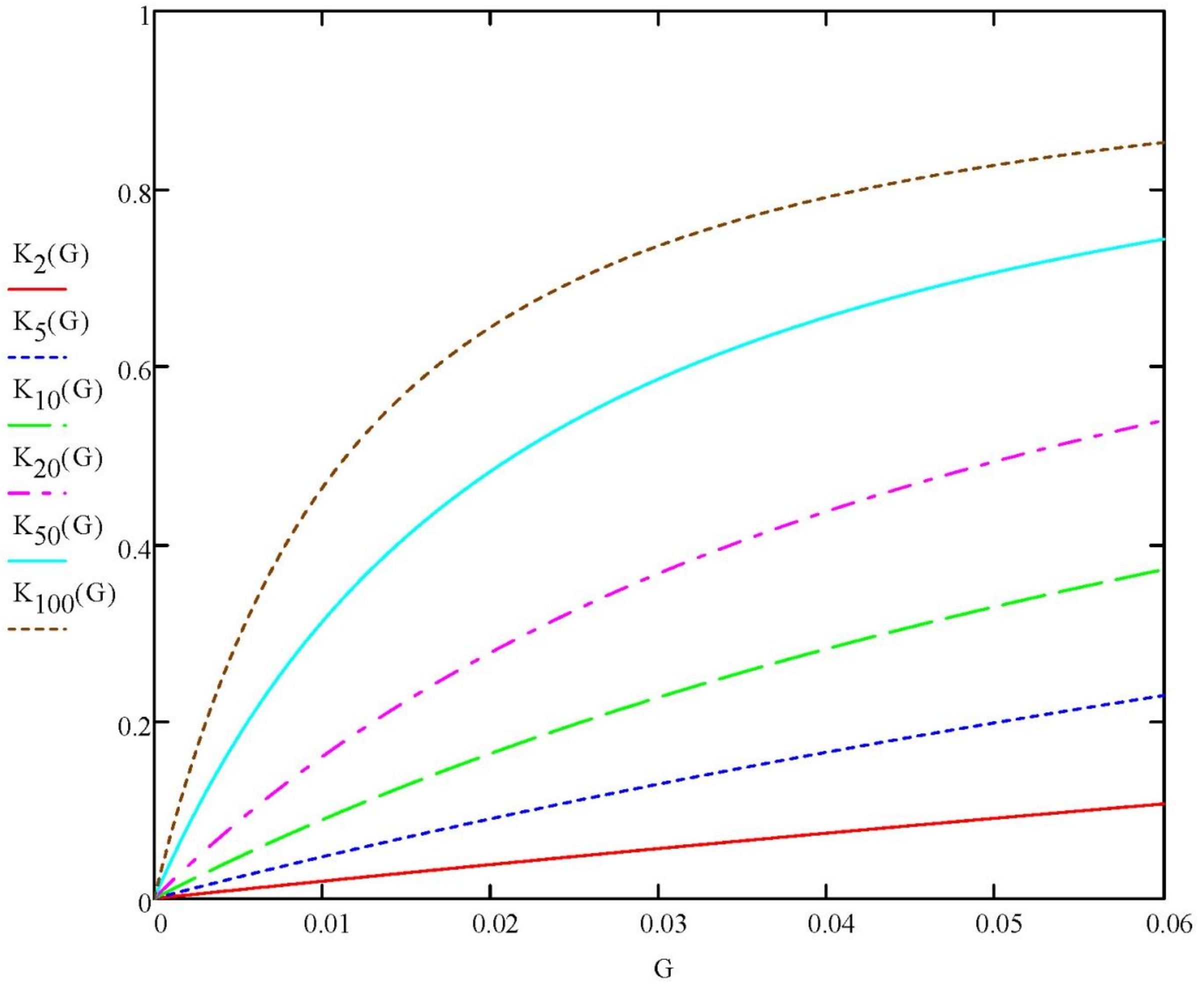

Figure S10: Calculated titration curves for K_{ass} = 2 up to K_{ass} = 100. The change in CIS ($\Delta\delta$, in ppm) is plotted against the concentration of the guest (G, in M^{-1}).

In the normalizing procedure, the CIS at 5 eq. was divided by the CIS at 20 eq. and the CIS at 20 eq. was divided by itself.

$$eq := \begin{pmatrix} 0 \\ 5 \\ 20 \end{pmatrix}$$

$$K_{2n} := \begin{bmatrix} 0 \\ \left(\dfrac{K_2(5\cdot H)}{K_2(20\cdot H)}\right) \\ 1 \end{bmatrix} \quad K_{5n} := \begin{bmatrix} 0 \\ \left(\dfrac{K_5(5\cdot H)}{K_5(20\cdot H)}\right) \\ 1 \end{bmatrix} \quad K_{10n} := \begin{bmatrix} 0 \\ \left(\dfrac{K_{10}(5\cdot H)}{K_{10}(20\cdot H)}\right) \\ 1 \end{bmatrix}$$

$$K_{20n} := \begin{bmatrix} 0 \\ \left(\dfrac{K_{20}(5\cdot H)}{K_{20}(20\cdot H)}\right) \\ 1 \end{bmatrix} \quad K_{50n} := \begin{bmatrix} 0 \\ \left(\dfrac{K_{50}(5\cdot H)}{K_{50}(20\cdot H)}\right) \\ 1 \end{bmatrix} \quad K_{100n} := \begin{bmatrix} 0 \\ \left(\dfrac{K_{100}(5\cdot H)}{K_{100}(20\cdot H)}\right) \\ 1 \end{bmatrix}$$

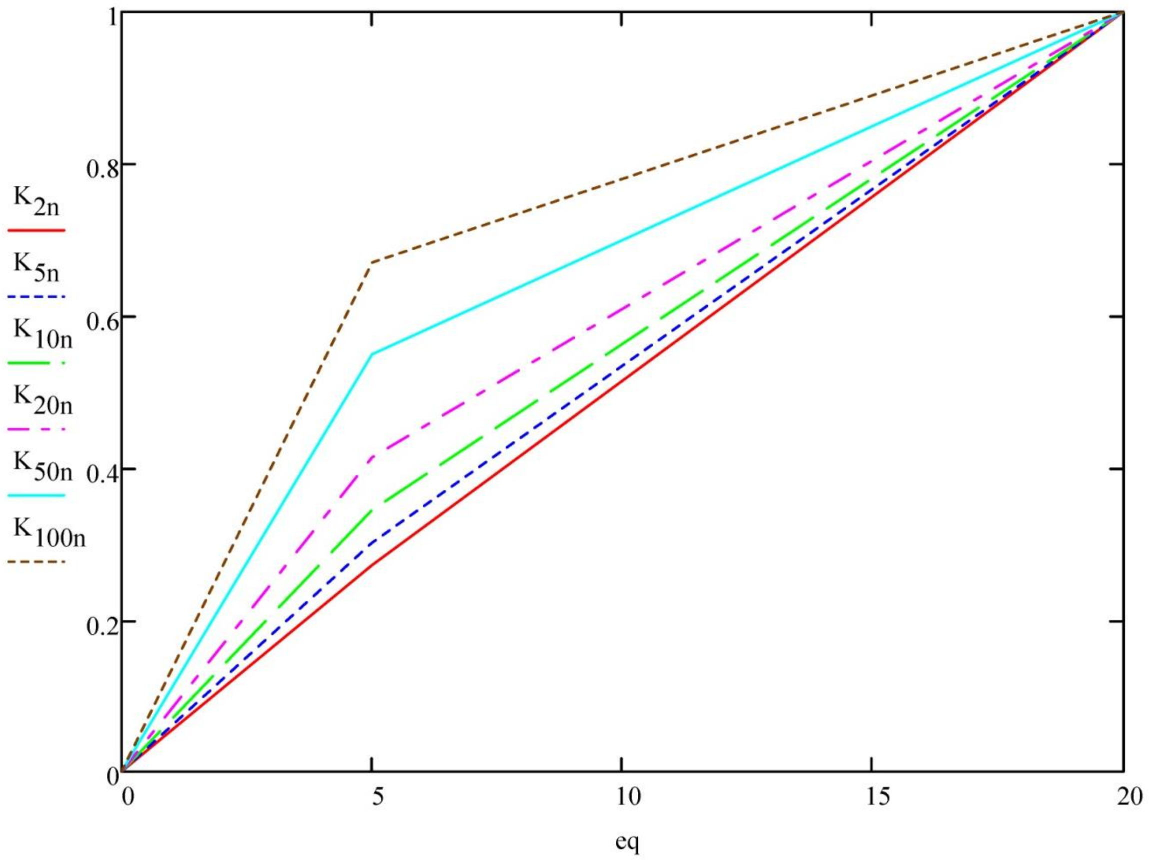

Figure S11: The resulting graph shows the simple correlation between the association constant and the deviation from the diagonal (0,0 → 20,1). The normalized change in CIS ($\Delta\delta_n$, in ppm) is plotted against the number of equivalents of the guest.

In a second test, different titration curves with different $\Delta\delta_{max}$ (0.3, 1, 3 ppm) were simulated at a constant K_{ass} = 20.

$$K := 20$$

$$H := 0.003 \qquad G := 0, 0.0001 .. 0.06$$

K = association constant (M^{-1})
G = concentration guest (M^{-1})
H = concentration host (M^{-1})
$\Delta\delta_{max}$ =shift(G_{sat}) - shift (G_0) (ppm)

$$\Delta\delta_{max} := 0.3$$

$$\Delta\delta_{0.3}(G) := \left[\left[\frac{\Delta\delta_{max}}{(2\cdot H)}\right]\cdot\left[\left[G + H + \left(\frac{1}{K}\right)\right] - \left[\left[(G-H)^2\right] + \left(2\cdot\frac{G}{K}\right) + \left(2\cdot\frac{H}{K}\right) + \left[\frac{1}{(K)^2}\right]\right]^{0.5}\right]\right]$$

$$\Delta\delta_{max} := 1$$

$$\Delta\delta_{1}(G) := \left[\left[\frac{\Delta\delta_{max}}{(2\cdot H)}\right]\cdot\left[\left[G + H + \left(\frac{1}{K}\right)\right] - \left[\left[(G-H)^2\right] + \left(2\cdot\frac{G}{K}\right) + \left(2\cdot\frac{H}{K}\right) + \left[\frac{1}{(K)^2}\right]\right]^{0.5}\right]\right]$$

$$\Delta\delta_{max} := 3$$

$$\Delta\delta_{3}(G) := \left[\left[\frac{\Delta\delta_{max}}{(2\cdot H)}\right]\cdot\left[\left[G + H + \left(\frac{1}{K}\right)\right] - \left[\left[(G-H)^2\right] + \left(2\cdot\frac{G}{K}\right) + \left(2\cdot\frac{H}{K}\right) + \left[\frac{1}{(K)^2}\right]\right]^{0.5}\right]\right]$$

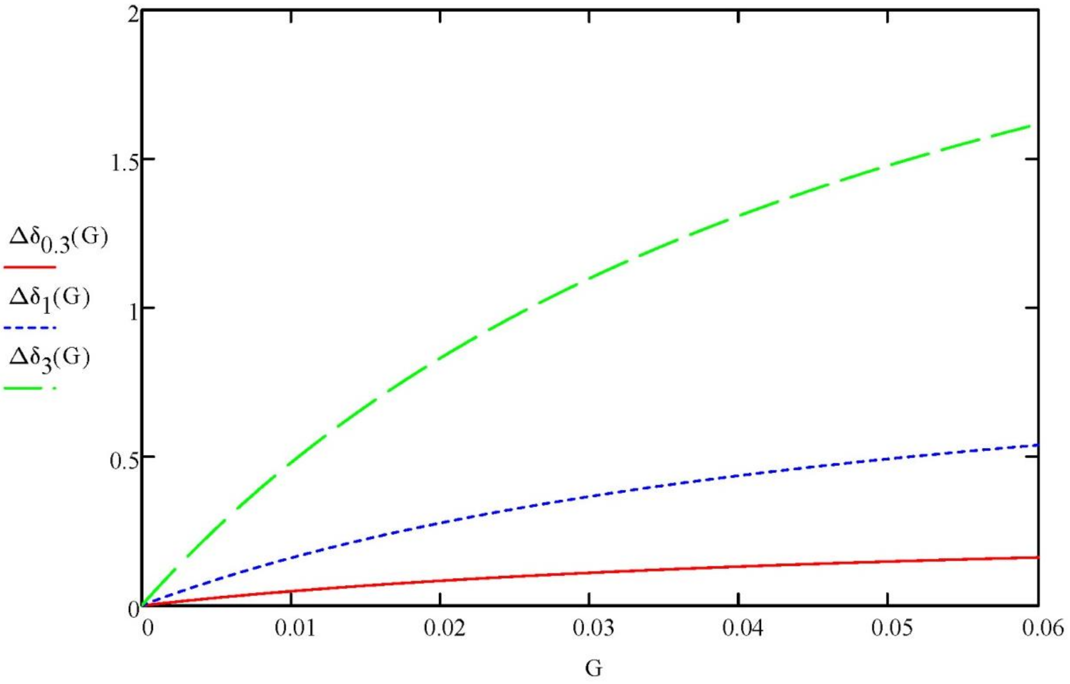

Figure S12: Calculated titration curves for different $\Delta\delta_{max}$ (0.3, 1, 3 ppm) at a constant association constant of 20 M^{-1}. The change in CIS ($\Delta\delta$, in ppm) is plotted against the concentration of the guest (G, in mol L^{-1}).

In the normalized procedure, the CIS at 5 eq. was divided by the CIS at 20 eq. and the CIS at 20 eq. was divided by itself.

$$q := \begin{pmatrix} 0 \\ 5 \\ 20 \end{pmatrix} \qquad \Delta\delta_{0.3n} := \begin{bmatrix} 0 \\ \left(\dfrac{\Delta\delta_{0.3}(5\cdot H)}{\Delta\delta_{0.3}(20\cdot H)} \right) \\ 1 \end{bmatrix} \quad \Delta\delta_{1n} := \begin{bmatrix} 0 \\ \left(\dfrac{\Delta\delta_{1}(5\cdot H)}{\Delta\delta_{1}(20\cdot H)} \right) \\ 1 \end{bmatrix} \quad \Delta\delta_{3n} := \begin{bmatrix} 0 \\ \left(\dfrac{\Delta\delta_{3}(5\cdot H)}{\Delta\delta_{3}(20\cdot H)} \right) \\ 1 \end{bmatrix}$$

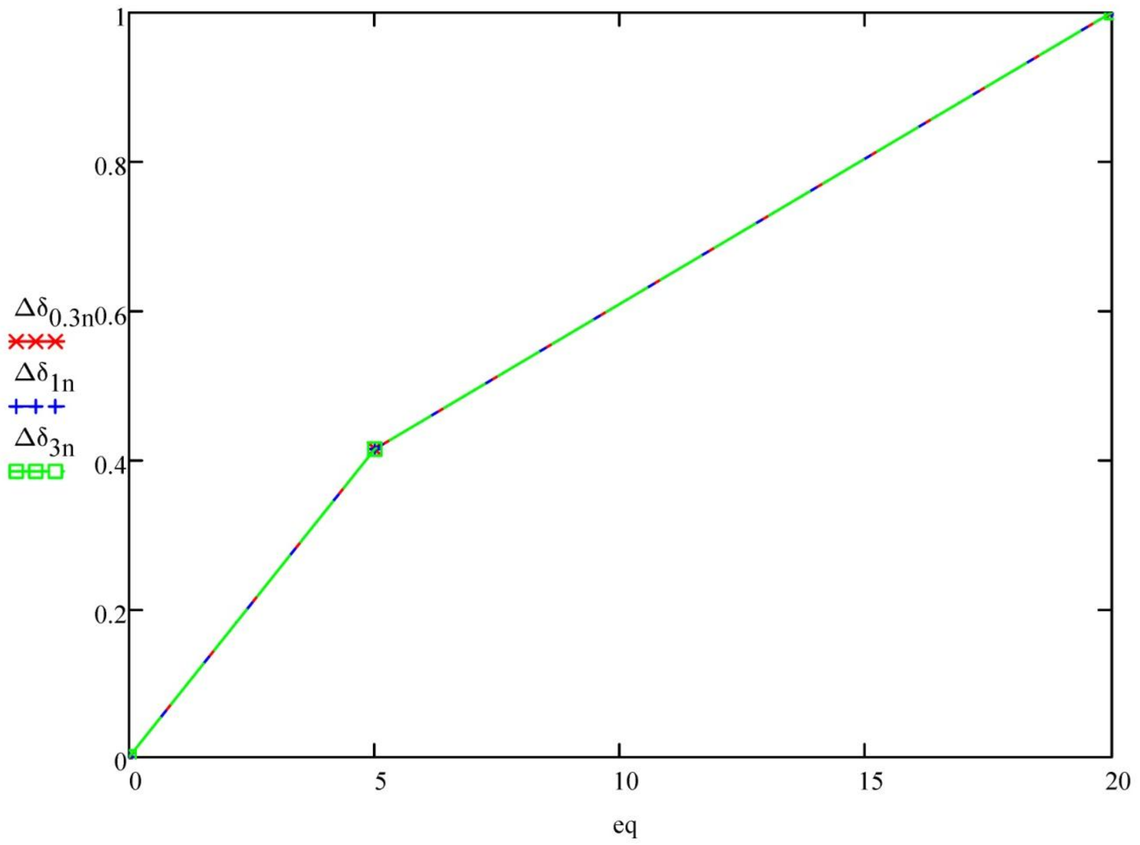

Figure S13: The resulting graph shows that the normalizing CIS method is not influenced by the maximum CIS of a titration experiment ($\Delta\delta_{max}$).

In an additional test, all six different association constants were permuted with these three different $\Delta\delta_{max}$ with results analogous to those already presented.

A cross-check of the normalized CIS method was carried out for a ^{1}H NMR titration with a small binding constant of K_{ass}= 37 M^{-1}, published in the literature [1,2].

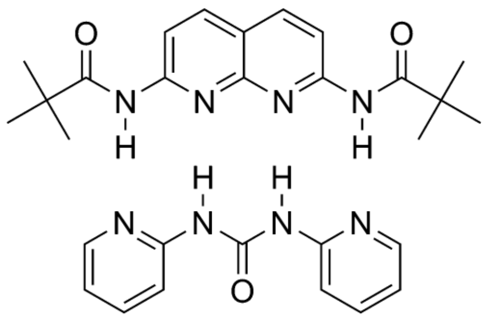

By the normalizing CIS method employing only two points, an approximate association constant of K_{ass} = ca. 40 M^{-1} was determined.

All calculations were carried out by using Mathcad® 15.0 (Parametric Technology Corporation).

¹H NMR titration of 1 with pyridine-*N*-oxide

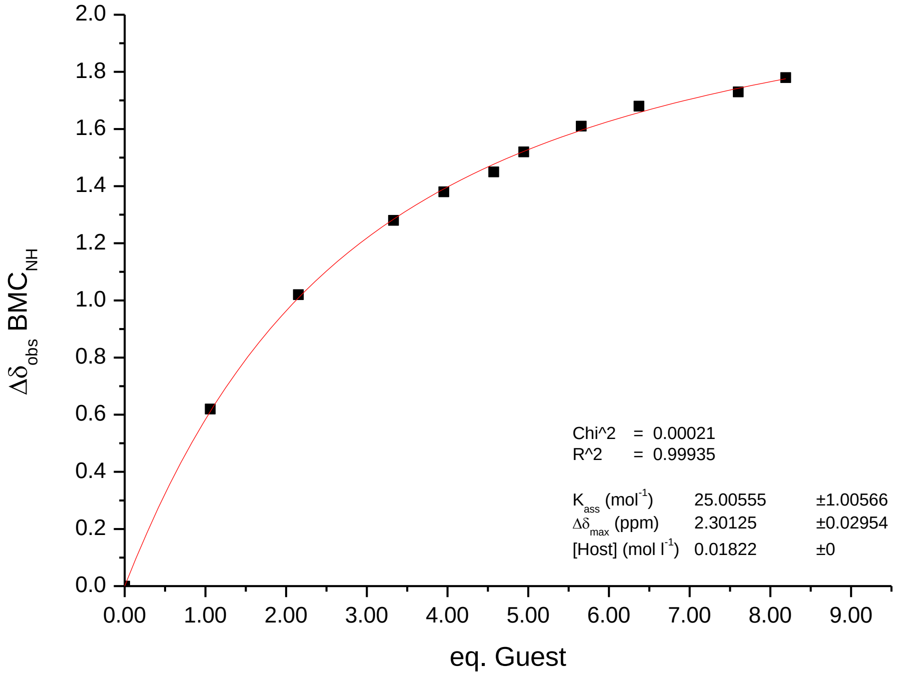

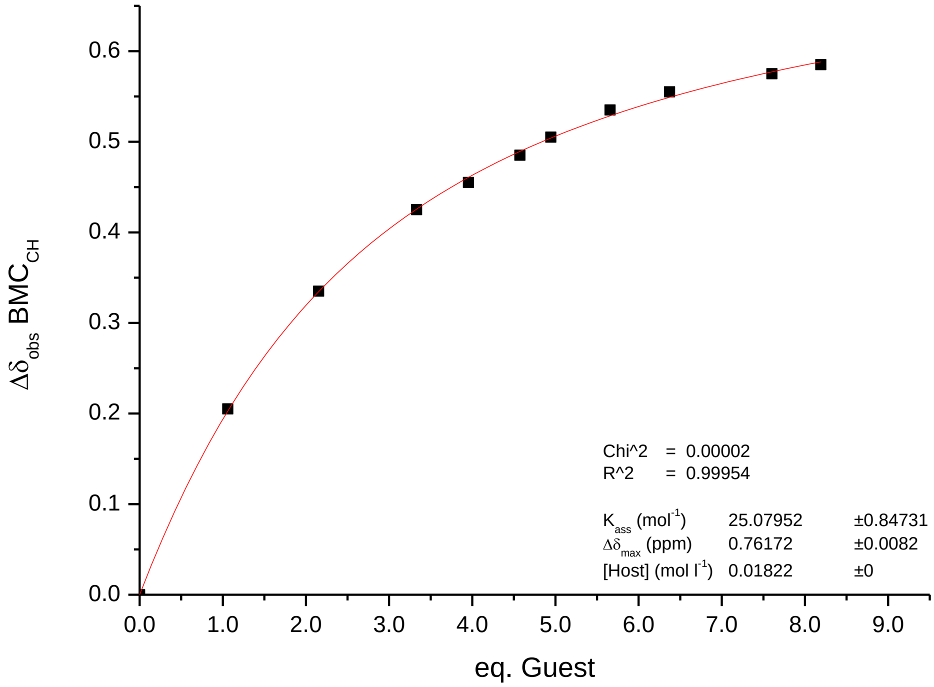

Figure S14: NMR titration of **1** and pyridine-*N*-oxide (500 MHz, 298 K, CD_2Cl_2). To a solution of **1** (7.72 mg in 600 µL CD_2Cl_2) was added pyridine-*N*-oxide (in CD_2Cl_2) in ten steps. Two signals were observed: amide NH (top) and the *endo*-CH (bottom), both resulting in K_{ass} = 25 mol^{-1} L.

^{1}H,^{1}H NOESY experiments

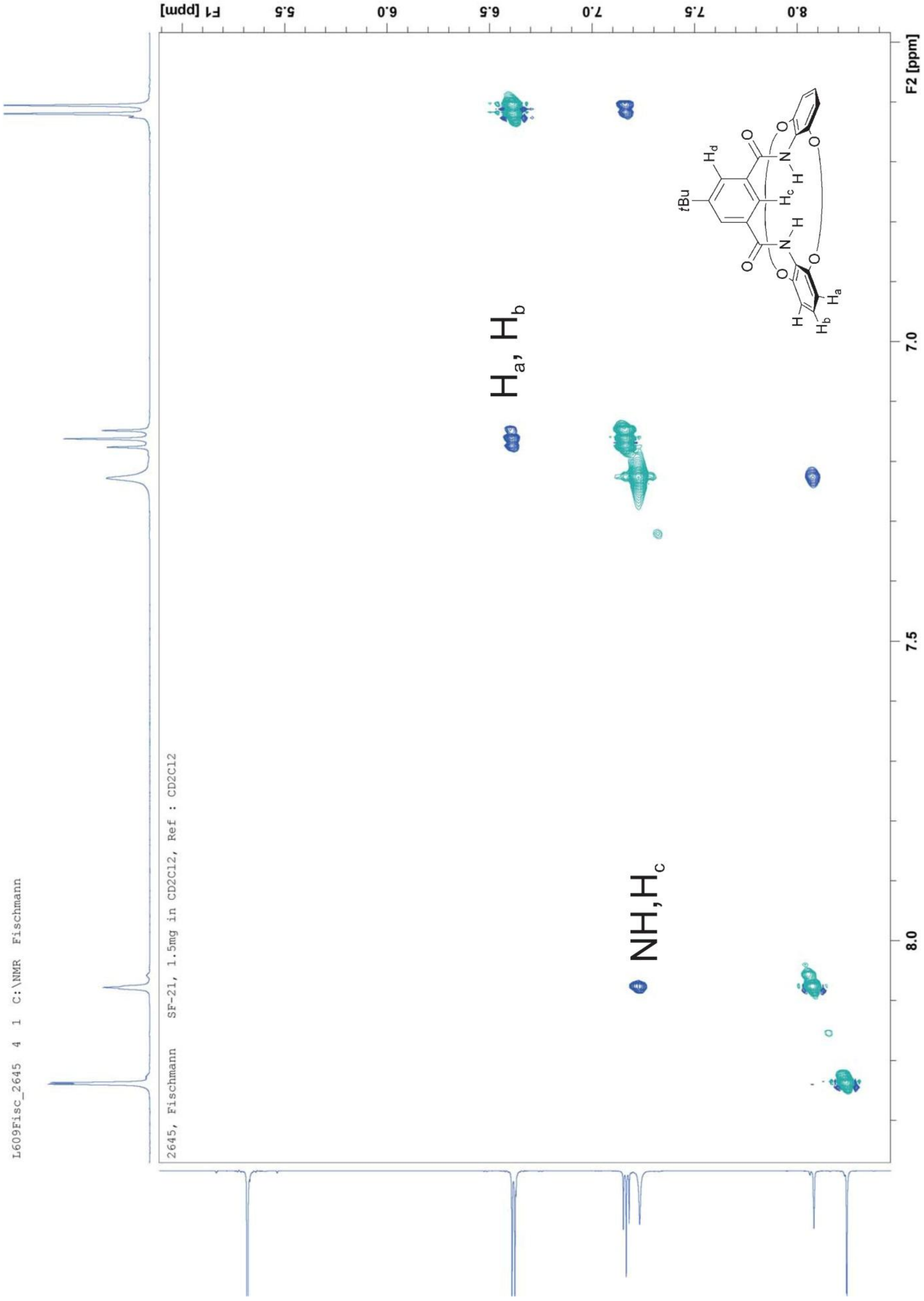

Figure S15: Bi-macrocycle **1**. NOESY experiment, selected signals (600 MHz, 298 K, CD_2Cl_2).

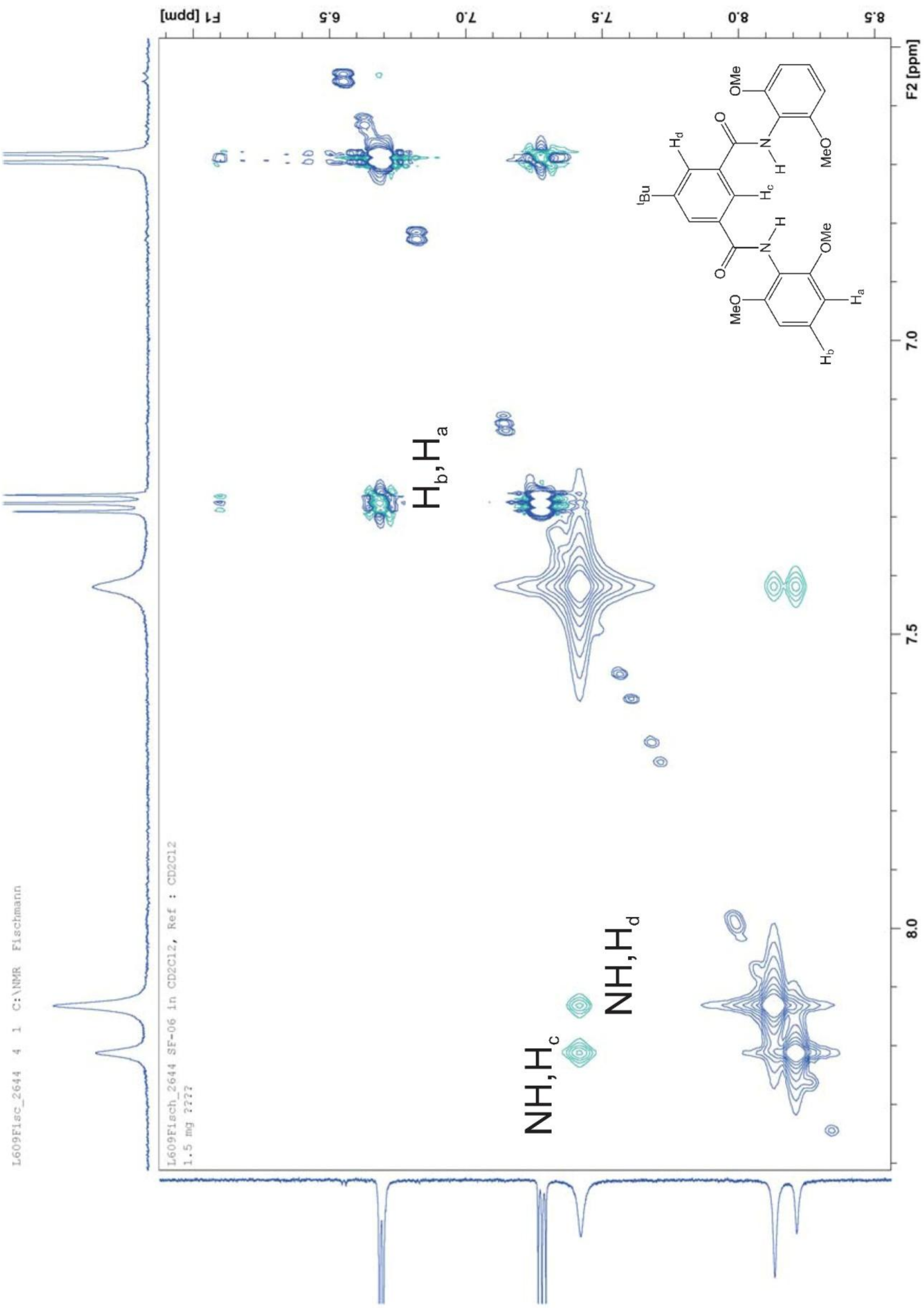

Figure S16: Isophthalamide **2**. NOESY experiment, selected signals (600 MHz, 298 K, CD_2Cl_2).

Transport experiments

Preparation of phospholipid vesicles: The solvent of a solution of 1-palmitoyl-2-oleoyl-*sn*-glycero-3-phosphocholine (POPC, Genzyme) in chloroform (20 mg/mL) was evaporated to leave a lipid film. This was then dried under vacuum for 12 h. The lipid film was rehydrated with a solution of sodium chloride (476 mM of NaCl, 10 mM of phosphate buffer, pH = 7.2) and shaken by vortex. The suspension was then subjected to nine freeze–thaw cycles and 29 extrusions through a 200 nm polycarbonate Nucleopore membrane by using a LiposoFast Basic extruder (Avestin) to obtain unilamellar vesicles with a mean diameter of 200 nm. Finally, the suspension was dialysed against a $NaNO_3$ solution (476 mM $NaNO_3$ and 10 mM phosphate buffer, pH = 7.2) to remove unencapsulated NaCl.

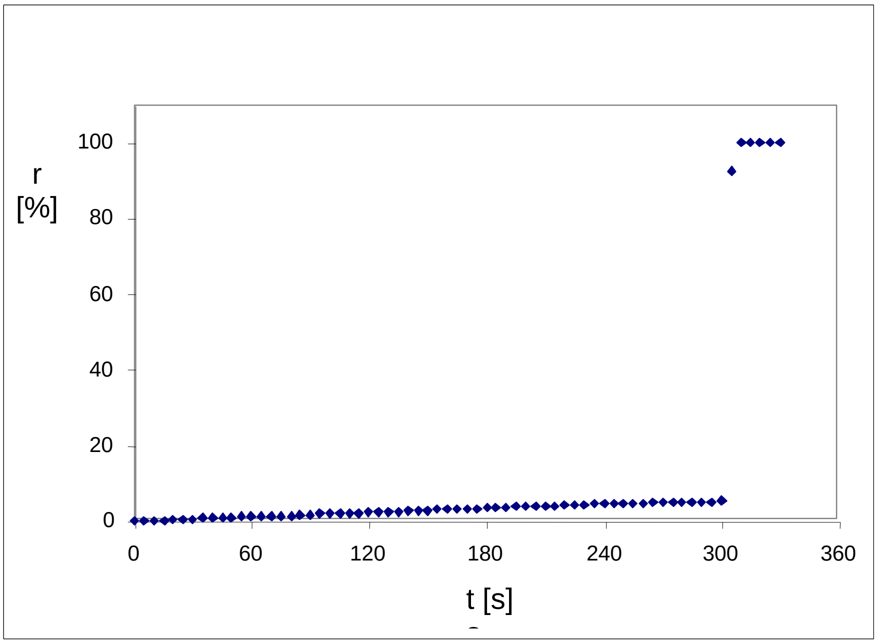

Figure S17: Chloride efflux upon addition of **2** (50 μM, 10 mol % carrier to lipid) to vesicles composed of 1-palmitoyl-2-oleoyl-*sn*-glycero-3-phosphocholine (POPC). The vesicles contained NaCl (476 mM NaCl and 10 mM phosphate buffer, pH 7.2) and were immersed in $NaNO_3$ (476 mM $NaNO_3$ and 10 mM phosphate buffer, pH 7.2). Once the electrode reading was stable the carrier was added and the chloride efflux was monitored for 5 min. At the end of the experiment, the vesicles were lysed with detergent to release all chloride ions and the resulting value was considered to represent a 100% release and used as such.

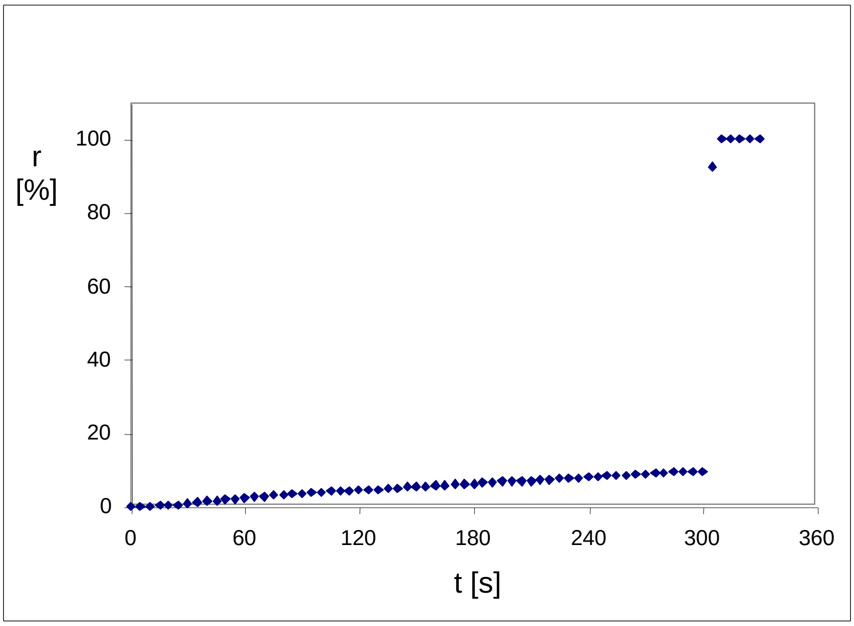

Figure S18: Chloride efflux upon addition of **1** (50 μM, 10 mol-% carrier to lipid) to vesicles composed of 1-palmitoyl-2-oleoyl-*sn*-glycero-3-phosphocholine (POPC). The vesicles contained NaCl (476 mM NaCl and 10 mM phosphate buffer, pH 7.2) and were immersed in $NaNO_3$ (476 mM $NaNO_3$ and 10 mM phosphate buffer, pH 7.2). Once the electrode reading was stable the carrier was added and the chloride efflux was monitored for 5 min. At the end of the experiment, the vesicles were lysed with detergent to release all chloride ions and the resulting value was considered to represent a 100% release and used as such.

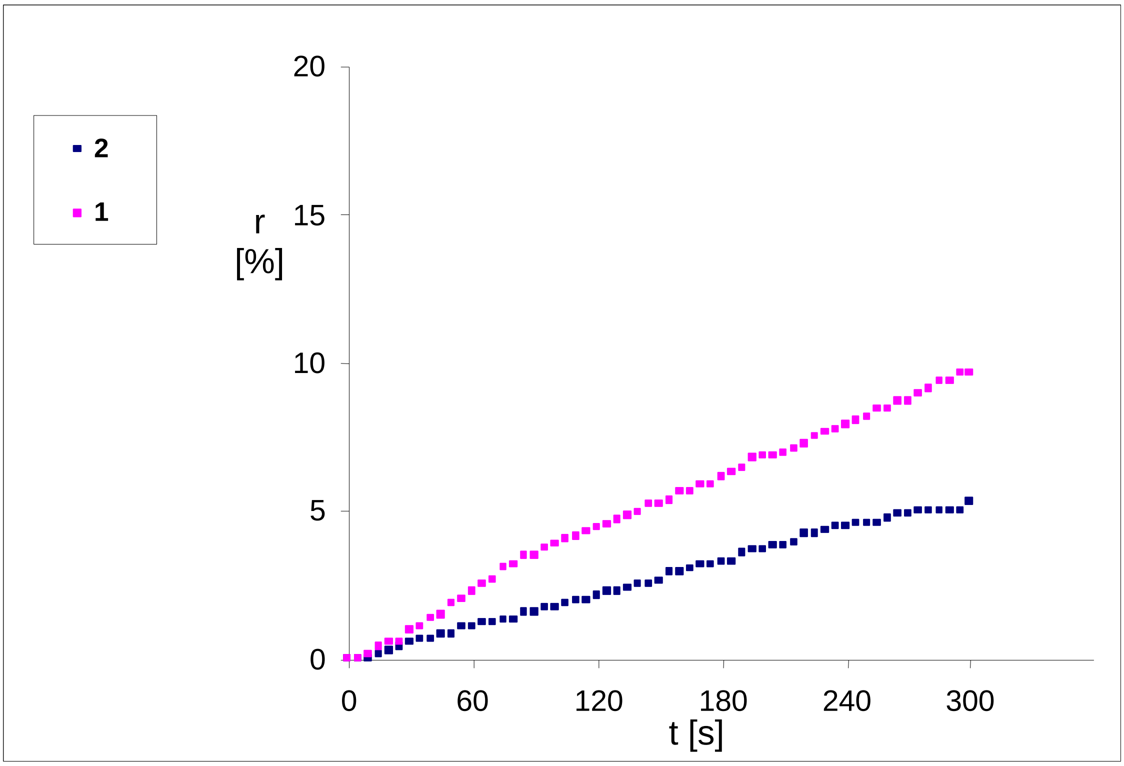

Figure S19: Comparison between chloride effluxes from chloride loaded liposomes promoted by the isophthalamides **1** and **2**.

References

1. Kühl, C.; Lüning, U. *Tetrahedron Lett.* **1998,** *39,* 5735–5738. doi:10.1016/S0040-4039(98)01200-3

2. Kühl, C. Ph.D. Thesis; Christian-Albrechts-Universität zu Kiel, 1998.
